# Australian Injury Comorbidity Indices (AICIs) to predict burden and readmission among hospital-admitted injury patients

**DOI:** 10.1186/s12913-021-06149-1

**Published:** 2021-02-15

**Authors:** Dasamal Tharanga Fernando, Janneke Berecki-Gisolf, Stuart Newstead, Zahid Ansari

**Affiliations:** 1 Monash University Accident Research Centre, Monash University, Clayton Campus, 21 Alliance Lane, Clayton, 3800 Victoria Australia; 2 Victorian Agency for Health Information, 50 Lonsdale Street, Melbourne, Victoria 3000 Australia

**Keywords:** Burden, Comorbidity, Cost, Index, Injury, Readmission

## Abstract

**Background:**

Existing comorbidity measures predict mortality among general patient populations. Due to the lack of outcome specific and patient-group specific measures, the existing indices are also applied to non-mortality outcomes in injury epidemiology. This study derived indices to capture the association between comorbidity, and burden and readmission outcomes for injury populations.

**Methods:**

Injury-related hospital admissions data from July 2012 to June 2014 (161,334 patients) for the state of Victoria, Australia were analyzed. Various multivariable regression models were run and results used to derive both binary and weighted indices that quantify the association between comorbidities and length of stay (LOS), hospital costs and readmissions. The new and existing indices were validated internally among patient subgroups, and externally using data from the states of New South Wales and Western Australia.

**Results:**

Twenty-four comorbidities were significantly associated with overnight stay, twenty-seven with LOS, twenty-eight with costs, ten with all-cause and eleven with non-planned 30-day readmissions. The number of and types of comorbidities, and their relative impact were different to the associations established with the existing Charlson Comorbidity Index (CCI) and Elixhauser Comorbidity Measure (ECM). The new indices performed equally well to the long-listed ECM and in certain instances outperformed the CCI.

**Conclusions:**

The more parsimonious, up to date, outcome and patient-specific indices presented in this study are better suited for use in present injury epidemiology. Their use can be trialed by hospital administrations in resource allocation models and patient classification models in clinical settings.

**Supplementary Information:**

The online version contains supplementary material available at 10.1186/s12913-021-06149-1.

## Background

Hospital-admitted injury patients can experience adverse outcomes during the course of the hospital stay, and comorbidities have the potential to increase that burden. Adverse outcomes include complications, extended hospital stay, readmission to hospital, discharge to long-term nursing care facilities and death. Previous research shows that among hospitalised patients, burden-related outcomes such as readmission to hospital [1–3], length of stay in hospital (LOS) [4, 5] and hospital costs [6–8] are associated with comorbidity. Therefore, comorbidities can *increase the likelihood* of adverse events occurring, and among injury patients, comorbidities may worsen their outcomes. The ability to quantify the effect of comorbidities for injury patients can assist in predicting outcomes in clinical settings and estimating injury burden in epidemiological research.

Currently there are several established methods for quantifying the association between comorbidities and outcomes. These include capturing the presence of at least one, each or a count of all comorbidities, and the use of comorbidity indices such as the Charlson Comorbidity Index (CCI) [9] and the Elixhauser Comorbidity Measure (ECM) [10]. The CCI allocates a weighted summed score for seventeen comorbid conditions while the ECM is a binary representation of thirty conditions. The CCI was originally derived in 1987 to assess the effect of comorbidity on mortality and is one of the most widely used. It was last updated in 2011 by Quan et al. [11]. The ECM was enhanced by van Walraven et al. in 2009 into a total score [12] and is less popular than the CCI. Some research considered the ECM as inconvenient due to the high number of comorbidities, arguing this may result in over-fitting [13].

Factors associated with outcomes for injury patients are different to those for general hospital-admitted patients [10, 13, 14]. Comorbidity measures should therefore consider the study population as well as the outcome. The type of data available, disease prevalence and clinical relevance also play an important role when deriving such measures. Reflecting this, using existing indices such as the CCI (derived for mortality) does not work well for other burden outcomes [6, 14].

The purpose of this study was: (1) to derive and validate new indices to establish the association between comorbidity, and readmission and burden-related outcomes, such as LOS and hospital costs, among hospital-admitted injury patients using Australian administrative datasets; and (2) to compare the performance of the new indices with the CCI and ECM.

## Methods

### Data sources

An analysis of existing morbidity data from the states of Victoria (Victorian Admitted Episodes Dataset (VAED)), New South Wales (NSW) (Admitted Patient Data Collection (APDC)) and Western Australia (WA) (Hospital Morbidity Data Collection (HMDC)) was carried out. The Victorian dataset was used for deriving injury comorbidity indices and the interstate datasets were used for external validation. Data provision and linkage were undertaken by the Centre for Victorian Data Linkage (CVDL) in Victoria, the Centre for Health Record Linkage (CHeReL) in NSW and the Data Linkage Branch (DLB) in WA.

These datasets capture all public and private hospital admissions, and contain patient demographics and morbidity information. The morbidity information includes 40 diagnosis fields for Victoria, 51 for NSW and 78 for WA, consisting of disease, injury and external cause data, coded to the ICD Tenth Revision, Australian Modifications (ICD-10-AM) [15]. Mortality data were extracted from (i) the Registrar of Births, Deaths and Marriages in NSW and (ii) Mortality Register in WA. Direct and indirect hospital costs for Victoria was sourced from the Victorian Cost Data Collection by CVDL and linked to the VAED.

### Data linkage

Data linkage (using patient-specific identifiers) was performed by CVDL using deterministic data linkage for the Victorian data while CHeReL used probabilistic matching techniques for NSW, and DLB in WA used a multi-faceted process which includes numerous automated and manual sub-processes. CVDL estimates the false positive rate to be between 0.5 to 1%, and the false negative match rates to be between 1 and 2% [16] and CHeReL estimates the false positives to be around 0.5% [17]. It is expected that the false negatives in the Western Australian Data Linkage System exceed the number of false positives, estimates for which are not attempted by the linkage unit [18].

### Case selection

Injury cases were identified and selected as those with records containing an ICD-10-AM diagnosis code in the range “S00” to “T75” or “T79” in the first appearing diagnosis field in the morbidity datasets; a practice commonly used for national reporting [19]. Case selection was further limited to index injury (i.e., the first injury record in the morbidity dataset for a patient during the study period) and residents belonging to each state. Consecutive records of inward transfers from other hospitals or statistical separations within the same hospital were considered to be part of one episode. Children less than 15 years of age were excluded when deriving the indices and validating; the rationale being that children differ to the rest of the cohort in terms of comorbidity prevalence.

#### The Victorian cohort

The comorbidity indices derivation cohort consisted of adult patients with an index injury admission between 01 July 2012 and 30 June 2014 (140,094 patients). They were followed up over a period of two years for subsequent hospital admissions.

#### The NSW and WA cohorts

The same selection and followup process was used as for the Victorian cohort using the APDC (201,791 patients) and HMDC (71,771 patients). These patients were also followed up in the mortality data to identify patients for censoring; those who died within 30 days of hospital-discharge did not have a possibility for readmission and were excluded from analysis pertaining to readmissions within 30 days [20, 21].

### Coding of outcomes, factors and comorbidities

#### Outcomes

Two outcomes related to LOS, one cost outcome and two outcomes related to readmissions were chosen for index derivation, some details of which has been published before [22].

The LOS days in the morbidity datasets were re-coded as (1) a binary code “0” for those who were discharged on the same day and “1” for those who stayed overnight, and (2) the number of days in hospital as a continuous variable for the index episode for those who stayed at least overnight. Case selection for the second LOS outcome was limited to those who stayed less than 1 month in the index episode (92% of cases).

Hospital costs were recorded in Australian dollars (AUD), and were available for Victoria from July 2012 to June 2015 only for those admitted to public hospitals. Costs were standardized to 2012 dollar values using the Australian Consumer Price Index [23]. Due to its skewness it was log transformed for analysis.

Two forms of readmissions (any-cause and non-planned) were coded, both with a binary outcome: “1” for patients readmitted within 30 days and “0” otherwise, limited to the first occurring readmission. Patients who died in hospital and those who left against medical advice were excluded.

#### Factors

The factors considered were age, sex, body-region, injury type, injury severity, SEIFA (Socio Economic Indexes For Areas), country of birth, geographic region and comorbidity. The coding is similar to that used in our previous studies (Fernando et.al. 2019 & Fernando et al., 2020) [22, 24]. Injury severity was assessed using the ICD-based Injury Severity Score (ICISS) [25]. Using the *worst injury* method [26], a serious injury was considered to be one with an ICISS less than or equal to 0.941 (survival probability of 94.1%) [27]. The survival risk ratios used in calculating the ICISS were provided by the National Injury Surveillance Unit [28].

#### Comorbidities

A combination of the CCI [9] and ECM [10] comorbidity groups per Quan et al. (2011) [29] and Sundararajan et al. [30] were used to select the list of comorbidities for study. Further details can be found in Fernando et al. (2019 & 2020) [22, 24].

### Statistical analysis

A negative binomial regression model for LOS, a linear regression model for log transformed costs and a logistic regression model for the binary outcomes (overnight stay, all-cause- and non-planned 30-day readmission) were fitted using multivariable regression. Socio-demographic variables and injury characteristics were entered in the baseline models.

Predictive power of the logistic regression models was assessed using discrimination (area under the receiver operating characteristic curve) and classification tables. The area under the curve (AUC) ranges from 0 to 1, with a value of under 0.7 representing poor discrimination and anything above that as good discrimination. Classification tables were derived on the basis of using a classification cut-off probability that maximized the combined sensitivity and specificity of the table based on the receiver operating characteristic curve. The adjusted R^2^ and McFadden’s R^2^ was used to determine predictive ability for the linear and negative binomial models.

After running the baseline models, comorbidity was added in various forms. Models were compared using the Akaike Information Criterion (AIC) [31]. A difference of less than 10 between two AICs indicates that the model with the additional factors provides no further improvement to the model fit. Using a backward elimination process starting with all thirty-one conditions in the model fitted as binary variables, a final model was derived which excludes comorbidities that no longer improve the model and were hence eliminated by the process.

The significance of interaction terms was tested using the AIC statistic (for model fit) and the improvement to the predictive power of models using the changes in (i) the AUC statistics for logistic models and (ii) adjusted R^2^ and McFadden’s R^2^ for the linear and negative binomial models. If both the AIC statistics change and the improvement to predictive powers were significant, then the interaction terms were retained.

The final model containing the *binary* representation of comorbidities significant to the outcome was retained. This model was given the generic name the “Australian Injury Comorbidity Index”. However, depending on the outcome of interest, an extension term describing the outcome is added to the generic name.

A *weighted* comorbidity index was derived using the final binary model, weights were computed for each comorbid condition using the following: resulting odds ratios (ORs) for each condition from logistic regression, incident rate ratios (IRRs) for the negative binomial regression; and the exponential of the beta coefficients for the linear regression model. The following rules were applied in allocating weights: the condition was dropped from the index if the weight < 1.2; 1.2 ≤ weight < 1.5 resulted in a score of 1; 1.5 ≤ weight < 2.5 = 2; 2.5 ≤ weight < 3.5 = 3 and so on. These weights were summed up to create the summed weighted score.

Two more indices were also derived: one parsimonious binary injury comorbidity index for burden and one for readmissions. The first was created using only conditions that were associated with LOS and cost, while the second was based on conditions associated with readmissions.

Finally, a validation of the newly derived indices was carried out. The indices were internally validated on patient subgroups and externally on NSW and WA datasets. For the internal validations, patient sub-groups were selected based on demographics and/or injury characteristics. The groups were children (< 15 years), adults (> = 65 years), males, females, adults with non-severe injuries, adults with intracranial injuries, adults with blunt trauma, adults with penetrating injuries and hip fracture patients aged 45 years and over. The validation models were the same baseline model identified for each outcome in Victoria with the addition of the binary index, the weighted index, the burden index, the readmission index, CCI, updated CCI and ECM to represent comorbidity. The models with various indices were assessed for predictive ability using the AUC and classification tables and adjusted R^2^s. Stata 14.0 (StataCorp) was used to analyze the data [32].

## Results

### Overview of the Victorian study population

Nearly a third (30.9%) of the population were 65 years and above, 55.3% were males and approximately 13% had serious injuries. More than half (59.6%) of the patients had a main injury to the extremities and the highest proportion of injuries were fractures (41.3%) (Table 1).

**Table 1:** Characteristics of the Victorian, NSW and WA study populations

	Patients admitted^a^ between July 2012 and June 2014, n (%)											
	Victoria				NSW				WA			
	n	%	At least one comorbidity (%)	Count of comorbidities, mean (95% CI)	n	%	At least one comorbidity (%)	Count of comorbidities, mean (95% CI)	n	%	At least one comorbidity (%)	Count of comorbidities, mean (95% CI)
Total patients	161,334				233,521				84,877			
**Age group (years)**												
0–14 years	21,240	13.2	1.4	0.01 (0.01–0.02)	31,730	13.6	1.5	0.02 (0.01–0.02)	13,106	15.4	1.9	0.02 (0.02–0.02)
15–24 years	23,213	14.4	9.9	0.12 (0.11–0.12)	33,888	14.5	11.0	0.13 (0.13–0.14)	13,676	16.1	15.3	0.18 (0.17–0.19)
25–44 years	36,262	22.5	13.0	0.16 (0.16–0.17)	51,737	22.2	13.5	0.17 (0.17–0.18)	22,617	26.6	18.6	0.23 (0.22–0.23)
45–64 years	30,799	19.1	19.3	0.25 (0.25–0.26)	44,501	19.1	18.3	0.25 (0.24–0.26)	16,344	19.3	21.6	0.29 (0.28–0.30)
65–84 years	31,390	19.5	35.9	0.54 (0.53–0.54)	44,948	19.2	33.2	0.51 (0.50–0.52)	12,598	14.8	36.0	0.55 (0.54–0.57)
85 and over	18,430	11.4	40.0	0.62 (0.60–0.63)	26,717	11.4	38.2	0.60 (0.59–0.62)	6536	7.7	41.5	0.64 (0.62–0.66)
**Sex**												
Male^b^	89,144	55.3	16.8	0.24 (0.23–0.24)	131,598	56.4	16.2	0.23 (0.23–0.24)	49,994	58.9	18.0	0.24 (0.24–0.25)
Female	72,190	44.7	23.5	0.33 (0.33–0.34)	101,923	43.6	22.7	0.33 (0.32–0.33)	34,883	41.1	24.0	0.33 (0.32–0.34)
**Injury severity**^c^												
Serious injury (ICISS< 0.941)	20,884	12.9	40.6	0.64 (0.63–0.65)	30,265	13.0	35.5	0.57 (0.56–0.59)	9566	11.3	38.3	0.60 (0.58–0.62)
Other injury (ICISS> = 0.941)	140,450	87.1	16.7	0.22 (0.22–0.23)	203,256	87.0	16.6	0.23 (0.23–0.23)	75,311	88.7	18.2	0.24 (0.23–0.24)
**Grouped body region**												
Head/face/neck	32,052	19.9	19.2	0.26 (0.25–0.27)	48,159	20.6	20.7	0.29 (0.28–0.29)	18,350	21.6	23.7	0.31 (0.30–0.32)
Trunk	20,730	12.8	25.0	0.36 (0.35–0.37)	29,199	12.5	23.1	0.34 (0.33–0.35)	9835	11.6	23.5	0.34 (0.33–0.35)
Upper extremity	54,549	33.8	9.8	0.12 (0.12–0.13)	77,389	33.1	9.4	0.12 (0.12–0.13)	27,833	32.8	10.9	0.14 (0.13–0.14)
Lower extremity	41,528	25.7	22.8	0.34 (0.33–0.35)	57,954	24.8	19.6	0.30 (0.30–0.31)	20,678	24.4	20.9	0.31 (0.30–0.32)
Multiple body regions	53	0.0	18.9	0.25 (0.09–0.40)	170	0.1	18.8	0.26 (0.17–0.35)	51	0.1	25.5	0.31 (0.15–0.47)
Unspecified body region	585	0.4	22.6	0.31 (0.26–0.37)	1247	0.5	22.3	0.34 (0.30–0.38)	376	0.4	22.1	0.30 (0.23–0.36)
Body region not relevant	11,837	7.3	47.7	0.66 (0.65–0.68)	19,403	8.3	45.5	0.65 (0.64–0.66)	7754	9.1	41.6	0.56 (0.55–0.58)
**Grouped injury type (First occurring)**												
Superficial injury	8055	5.0	23.8	0.34 (0.32–0.35)	14,875	6.4	22.6	0.33 (0.32–0.34)	4659	5.5	27.7	0.37 (0.35–0.39)
Open wound	22,398	13.9	14.8	0.19 (0.18–0.20)	35,691	15.3	16.9	0.23 (0.22–0.23)	12,623	14.9	20.6	0.27 (0.26–0.28)
Fracture	66,686	41.3	19.6	0.29 (0.28–0.29)	94,258	40.4	17.3	0.26 (0.26–0.27)	30,714	36.2	18.5	0.27 (0.26–0.27)
Dislocation, sprain & strain	10,989	6.8	8.2	0.10 (0.10–0.11)	13,330	5.7	6.6	0.09 (0.08–0.09)	6755	8.0	7.5	0.09 (0.08–0.10)
Injury to nerves & spinal cord	2038	1.3	10.4	0.13 (0.11–0.14)	2559	1.1	8.6	0.11 (0.10–0.13)	1147	1.4	11.1	0.14 (0.11–0.16)
Injury to blood vessels	1347	0.8	11.5	0.15 (0.12–0.17)	1096	0.5	15.0	0.19 (0.16–0.22)	650	0.8	18.5	0.23 (0.19–0.28)
Injury to muscle & tendon	8451	5.2	9.1	0.11 (0.10–0.12)	10,921	4.7	6.7	0.08 (0.08–0.09)	5423	6.4	10.1	0.12 (0.11–0.13)
Crushing injury	335	0.2	3.3	0.04 (0.01–0.06)	469	0.2	3.4	0.04 (0.02–0.06)	166	0.2	4.2	0.05 (0.01–0.08)
Traumatic amputation	1612	1.0	7.3	0.08 (0.07–0.10)	1622	0.7	6.8	0.08 (0.06–0.10)	747	0.9	7.9	0.09 (0.07–0.11)
Eye injury- excluding foreign body	512	0.3	16.4	0.22 (0.17–0.27)	919	0.4	17.3	0.22 (0.19–0.26)	308	0.4	19.5	0.23 (0.17–0.28)
Intracranial injury	6416	4.0	29.4	0.43 (0.41–0.45)	9355	4.0	28.1	0.42 (0.41–0.44)	3385	4.0	30.6	0.44 (0.41–0.47)
Injury to internal organs	1762	1.1	23.5	0.32 (0.29–0.35)	2198	0.9	22.5	0.32 (0.29–0.35)	1066	1.3	23.7	0.34 (0.29–0.38)
Foreign body	2733	1.7	11.3	0.15 (0.13–0.16)	3947	1.7	9.2	0.12 (0.10–0.13)	1486	1.8	9.6	0.12 (0.10–0.14)
Burns	1879	1.2	15.1	0.21 (0.18–0.23)	3244	1.4	8.8	0.12 (0.10–0.14)	1660	2.0	14.3	0.20 (0.17–0.22)
Other and unspecified injury	14,284	8.9	19.8	0.27 (0.26–0.28)	19,634	8.4	19.6	0.28 (0.27–0.29)	6334	7.5	22.8	0.30 (0.29–0.32)
Systemic-poisoning/toxic effects	10,536	6.5	49.5	0.68 (0.67–0.70)	17,162	7.3	47.8	0.67 (0.66–0.68)	6866	8.1	43.5	0.59 (0.57–0.60)
Other effects of external cause/complication	1301	0.8	32.5	0.54 (0.48–0.59)	2241	1.0	28.2	0.47 (0.43–0.50)	888	1.0	26.7	0.40 (0.35–0.45)
**Geographic region**												
Metropolitan Area	118,959	73.7	19.8	0.28 (0.27–0.28)	158,595	67.9	18.9	0.28 (0.27–0.28)	60,151	70.9	19.7	0.27 (0.27–0.28)
Regional Area	42,375	26.3	19.8	0.28 (0.27–0.29)	74,918	32.1	19.2	0.27 (0.26–0.27)	24,405	28.8	22.1	0.29 (0.28–0.30)
Unknown					8	0.0	*	0.25 (−0.07–0.57)	321	0.4	34.6	0.42 (0.35–0.50)
All-cause readmissions^d^	17,946	11.4	18.0	0.46 (0.45–0.47)	30,922	13.7	25.1	0.38 (0.37–0.39)	9930	12.1	30.6	0.45 (0.43–0.47)
Non-planned readmissions^d^	11,954	7.6	12.7	0.50 (0.48–0.51)	17,362	7.7	30.8	0.47 (0.46–0.49)	6130	7.5	36.1	0.53 (0.51–0.55)
LOS (median, IQR)	1 (1–3)		4 (1–17)	0.28 (0.27–0.28)	1 (1–4)		4 (1–15)	0.27 (0.27–0.28)	1 (1–3)		2 (1–10)	0.28 (0.27–0.28)
Overnight stay	110,337	68.4	24.7	0.36 (0.35–0.36)	165,056	70.7	23.3	0.34 (0.34–0.35)	58,886	69.4	22.9	0.33 (0.32–0.33)
Cost (mean, 95% CI)^e^	7457.3 (7370.1–7544.5)		14,157.5 (13,876.8–14,438.2)									

Notes: Small cell counts (frequency <5), and/or proportions resulting from them are represented by "*" to maintain confidentiality

^a^. Index injury admissions to all public and private hospitals, limited to residents within the relevant state

^b^. Intersex/intermediate/unstated sex patient counts less than 5 added to the majority sex group to protect confidentiality

^c^. Worst injury method-ICD-based Injury Severity Score less than or equal to 0.941 considered as serious injury

^d^. Based on 157,866 patients for Victoria, 225,047 for NSW and 82,212 for WA (excludes patients who died or left against medical advice)

^e^. Based on 123,207 patients with complete cost data

The median LOS was 1 day (IQR 1–3) for the entire cohort while those with at least one comorbidity had a higher median LOS of 4 days (IQR 1–17). Over two-thirds required an overnight stay (68.4%). The mean hospital cost was AUD 7457.3 (95% CI 7370.1 − 7544.5) (based on 123,207 episodes of care with complete cost data), while the mean cost for those with at least one comorbidity was nearly double (AUD 14,157.5 (95% CI 13,876.8 -14,438.2)). Excluding patients who died or left against medical advice, 11.4% had at least one any-cause and 7.6% one non-planned readmission to a hospital within 30 days of being discharged for the index admission. These readmissions increased to 18 and 12.7% respectively for those with at least one comorbidity. Overall, comorbidity increased with age, was higher among females, and higher among patients with serious-injuries. An overview of the NSW and WA study populations is also presented in Table 1, and details of all three populations in Appendix Table A1.

### Multivariable regression modelling

#### Comorbidity in the Victorian study population (adults)

Alcohol dependence, cardiac arrhythmia, dementia, depression, diabetes, hypertension without complications and renal disease were the most commonly recorded comorbidities among injured adults (> 15 year of age) (Table 2). After adjusting for baseline variables such as demographics, and injury characteristics including injury severity, the comorbidities associated with a high burden on the system (LOS and costs) were not the same comorbidities that were associated with high readmission rates.

**Table 2:** Presence of comorbidity and the association with burden and readmission outcomes (Victoria, age 15 years and over)

Comorbidity	Index admission LOS (*n* = 140,094)				Index hospital admission cost (*n* = 104,609)	
	n (%)	Index LOS (mean, 95% CI)	Adjusted OR^a,b ^(95% CI)	IRR^c,d^ (95% CI)	Cost^e^ (mean, 95% CI)	Adjusted beta coefficient^a ^(95% CI)
HIV/AIDS	54 (0.0)	11.1 (5.0–17.1)	1.05 (0.53–2.11)	1.11 (0.85–1.44)	13,064.9 (6672.5–19,457.4)	0.40 (0.12–0.68)
Alcohol dependence	6425 (4.6)	7.1 (6.6–7.6)	1.38 (1.29–1.48)	1.17 (1.14–1.20)	8927.3 (8464.9–9389.7)	0.20 (0.17–0.23)
Drug dependence	1496 (1.1)	8.1 (7.0–9.2)	2.34 (2.01–2.72)	1.56 (1.49–1.64)	11,228.8 (10,195.1–12,262.4)	0.47 (0.41–0.52)
Any malignancy	706 (0.5)	21.2 (19.5–22.8)	6.15 (3.09–12.22)	1.56 (1.40–1.73)	23,110.8 (21,132.8–25,088.8)	0.65 (0.52–0.78)
Blood loss anemia	170 (0.1)	24.7 (21.0–28.5)	5.56 (1.71–18.08)	1.40 (1.20–1.63)	29,192.2 (24,390.9–33,993.4)	0.61 (0.44–0.77)
Cardiac arrhythmias	3775 (2.7)	19.0 (18.2–19.8)	2.44 (2.14–2.79)	1.32 (1.28–1.36)	20,572.8 (19,549.5–21,596.0)	0.48 (0.44–0.51)
Cerebrovascular disease	602 (0.4)	24.2 (21.0–27.5)	1.74 (1.08–2.79)	1.18 (1.08–1.29)	23,691.7 (21,388.4–25,995.0)	0.19 (0.08–0.30)
Chronic pulmonary disease	1302 (0.9)	22.8 (21.3–24.2)	6.24 (4.22–9.22)	1.46 (1.38–1.54)	26,770.7 (24,643.6–28,897.8)	0.57 (0.51–0.64)
Coagulopathy	1133 (0.8)	18.9 (17.1–20.8)	3.73 (2.79–4.98)	1.43 (1.35–1.51)	22,255.5 (19,748.4–24,762.6)	0.49 (0.43–0.56)
Congestive heart failure	1257 (0.9)	24.8 (23.5–26.2)	6.84 (4.14–11.30)	1.27 (1.20–1.34)	26,944.0 (25,240.2–28,647.9)	0.38 (0.31–0.44)
Deficiency anemias	578 (0.4)	24.1 (21.9–26.2)	8.56 (4.65–15.75)	1.62 (1.50–1.76)	22,380.0 (20,429.5–24,330.5)	0.66 (0.56–0.75)
Dementia	2889 (2.1)	16.2 (15.5–16.9)	1.99 (1.70–2.34)	1.05 (1.02–1.09)	16,671.0 (15,968.2–17,373.8)	0.16 (0.11–0.20)
Depression	2966 (2.1)	9.2 (8.6–9.9)	3.25 (2.89–3.65)	1.93 (1.86–2.01)	10,987.7 (10,125.5–11,849.9)	0.63 (0.59–0.67)
Diabetes with chronic complications	4243 (3.0)	18.6 (17.9–19.4)	1.42 (1.26–1.60)	1.27 (1.23–1.31)	19,104.6 (18,238.8–19,970.5)	0.26 (0.22–0.30)
Diabetes without complications	8969 (6.4)	12.0 (11.5–12.4)	1.20 (1.13–1.28)	1.12 (1.09–1.14)	12,223.6 (11,710.2–12,737.0)	0.10 (0.07–0.12)
Hemiplegia/paraplegia	538 (0.4)	25.7 (22.3–29.1)	4.14 (2.51–6.83)	1.61 (1.47–1.78)	25,975.1 (22,961.5–28,988.6)	0.60 (0.49–0.71)
Hypertension complicated	54 (0.0)	25.4 (17.6–33.1)	O	1.41 (1.09–1.82)	28,041.4 (20,243.3–35,839.6)	0.48 (0.20–0.76)
Hypertension uncomplicated	4510 (3.2)	21.5 (20.7–22.2)	2.45 (2.11–2.84)	1.26 (1.22–1.30)	22,582.5 (21,702.8–23,462.3)	0.41 (0.37–0.45)
Hypothyroidism	152 (0.1)	21.3 (17.3–25.3)	21.00 (2.89–152.81)	1.72 (1.48–1.99)	22,116.8 (17,862.1–26,371.5)	0.56 (0.38–0.74)
Metastatic solid tumor	380 (0.3)	20.4 (18.3–22.5)	1.39 (0.54–3.57)	1.32 (1.15–1.52)	22,163.4 (19,616.7–24,710.1)	0.16 (−0.02–0.33)
Mild liver disease	1167 (0.8)	10.5 (9.4–11.7)	1.63 (1.37–1.93)	1.27 (1.20–1.34)	13,166.4 (11,933.4–14,399.4)	0.31 (0.25–0.38)
Moderate or severe liver disease	116 (0.1)	20.2 (15.1–25.3)	20.59 (2.83–149.95)	1.83 (1.54–2.19)	25,608.6 (19,508.8–31,708.4)	0.81 (0.60–1.01)
Myocardial infarction	248 (0.2)	23.9 (21.0–26.8)	2.78 (1.11–6.96)	0.96 (0.85–1.09)	27,805.7 (24,266.1–31,345.4)	0.24 (0.10–0.38)
Obesity	229 (0.2)	27.9 (22.6–33.2)	4.39 (2.26–8.54)	2.10 (1.85–2.39)	29,539.4 (23,323.0–35,755.7)	0.80 (0.65–0.95)
Peptic ulcer disease	84 (0.1)	20.3 (14.4–26.2)	1.96 (0.90–4.29)	1.55 (1.25–1.92)	23,885.5 (15,681.1–32,089.8)	0.66 (0.40–0.91)
Peripheral vascular disease	702 (0.5)	17.2 (15.4–19.1)	3.10 (2.34–4.11)	1.88 (1.76–2.02)	19,444.2 (17,341.6–21,546.9)	0.75 (0.66–0.83)
Psychoses	568 (0.4)	20.2 (17.2–23.1)	5.54 (3.96–7.74)	3.14 (2.91–3.39)	22,213.8 (19,103.3–25,324.3)	1.04 (0.95–1.13)
Pulmonary circulation disorders	133 (0.1)	33.6 (28.0–39.2)	9.06 (1.24–66.43)	1.81 (1.52–2.16)	39,206.1 (30,612.7–47,799.5)	0.79 (0.58–0.99)
Renal disease including renal failure	3402 (2.4)	22.3 (21.5–23.2)	1.54 (1.29–1.84)	1.16 (1.11–1.20)	22,865.3 (21,879.6–23,851.0)	0.20 (0.15–0.24)
Rheumatic disease including some other connective tissue disorders	177 (0.1)	25.4 (21.7–29.1)	31.75 (4.41–228.88)	1.93 (1.66–2.23)	27,277.3 (22,034.9–32,519.7)	0.84 (0.66–1.02)
Valvular disease	326 (0.2)	27.9 (24.8–31.0)	5.23 (2.27–12.02)	1.36 (1.22–1.52)	28,473.9 (24,958.1–31,989.7)	0.41 (0.28–0.55)
Comorbidity	Readmissions (*n* = 136,670)					
	n (%)	All-cause 30-day readmission			Non-planned 30-day readmission	
	beta coefficient^a^ (95% CI)	% with condition that had the outcome		Adjusted OR^a^ (95% CI)	% with condition that had the outcome	Adjusted OR^a^ (95% CI)
HIV/AIDS	53 (0.0)	20.8		1.50 (0.76–2.95)	20.8	2.57 (1.31–5.06)
Alcohol dependence	6119 (4.5)	15.3		1.22 (1.13–1.31)	11.4	1.34 (1.23–1.46)
Drug dependence	1350 (1.0)	16.0		1.27 (1.09–1.48)	12.9	1.45 (1.23–1.72)
Any malignancy	561 (0.4)	44.0		3.45 (2.71–4.40)	20.7	1.64 (1.21–2.23)
Blood loss anemia	159 (0.1)	22.0		1.20 (0.81–1.77)	14.5	1.09 (0.69–1.72)
Cardiac arrhythmias	3480 (2.5)	18.2		1.06 (0.97–1.16)	13.4	1.10 (0.99–1.22)
Cerebrovascular disease	545 (0.4)	19.1		1.08 (0.83–1.40)	14.3	1.08 (0.81–1.45)
Chronic pulmonary disease	1176 (0.9)	23.0		1.40 (1.21–1.61)	18.2	1.60 (1.37–1.86)
Coagulopathy	1032 (0.8)	23.5		1.43 (1.23–1.66)	18.1	1.53 (1.30–1.81)
Congestive heart failure	1048 (0.8)	24.0		1.20 (1.03–1.40)	19.3	1.34 (1.14–1.58)
Deficiency anemias	550 (0.4)	24.0		1.28 (1.05–1.58)	18.7	1.42 (1.13–1.77)
Dementia	2663 (1.9)	15.5		0.89 (0.79–0.99)	13.3	1.12 (1.00–1.26)
Depression	2877 (2.1)	18.8		1.36 (1.23–1.52)	11.8	1.14 (1.01–1.30)
Diabetes with chronic complications	3978 (2.9)	23.9		1.24 (1.13–1.35)	16.9	1.29 (1.16–1.43)
Diabetes without complications	8655 (6.3)	17.2		1.18 (1.11–1.25)	12.1	1.21 (1.13–1.30)
Hemiplegia/paraplegia	494 (0.4)	18.6		1.17 (0.89–1.54)	13.8	1.20 (0.88–1.63)
Hypertension complicated	42 (0.0)	40.5		1.89 (1.01–3.55)	21.4	1.30 (0.61–2.76)
Hypertension uncomplicated	4165 (3.0)	22.0		1.05 (0.96–1.15)	16.0	1.13 (1.02–1.25)
Hypothyroidism	148 (0.1)	17.6		0.93 (0.61–1.44)	14.9	1.15 (0.73–1.83)
Metastatic solid tumor	287 (0.2)	46.3		1.37 (0.98–1.92)	22.6	1.50 (0.99–2.26)
Mild liver disease	1055 (0.8)	21.5		1.65 (1.41–1.93)	17.3	1.84 (1.55–2.18)
Moderate or severe liver disease	90 (0.1)	30.0		1.45 (0.90–2.32)	23.3	1.41 (0.84–2.36)
Myocardial infarction	197 (0.1)	21.8		1.08 (0.76–1.53)	18.3	1.21 (0.83–1.76)
Obesity	219 (0.2)	20.1		1.34 (0.95–1.89)	16.4	1.59 (1.10–2.31)
Peptic ulcer disease	70 (0.1)	17.1		0.96 (0.51–1.82)	7.1	0.54 (0.21–1.36)
Peripheral vascular disease	673 (0.5)	19.5		1.37 (1.12–1.67)	12.9	1.30 (1.03–1.64)
Psychoses	536 (0.4)	21.6		1.64 (1.33–2.03)	15.1	1.56 (1.22–1.99)
Pulmonary circulation disorders	120 (0.1)	20.8		0.94 (0.59–1.49)	15.0	0.89 (0.53–1.51)
Renal disease including renal failure	3042 (2.2)	30.0		1.92 (1.73–2.12)	20.2	1.56 (1.39–1.76)
Rheumatic disease including some other connective tissue disorders	168 (0.1)	22.0		1.39 (0.95–2.02)	15.5	1.46 (0.95–2.24)
Valvular disease	290 (0.2)	19.3		1.03 (0.76–1.39)	14.5	1.08 (0.77–1.51)

Notes

^a^ Adjusted with baseline model - see Table 3 footnotes for baseline model details

^b^ Outcome=at least one overnight stay

^c^ Patients with same day separations (n=40819) excluded from the 140094 (adult patients) (n=99275)

^d^ Outcome=LOS for those who had at least one overnight stay but <= 30 days (n=8472 patients excluded from 99275)

^e^ Based on patients with complete cost data

^f^ O=Omitted as all cases had the outcome

#### Baseline models

Results for the baseline models are presented in Table 3 (model *i*). The baseline factors differ for each outcome (see stepwise breakdown of factor inclusion in Appendix Table A2). Age, sex and injury characteristics all improved the model fit for all baseline models except for all-cause readmissions, where adding sex and injury severity did not improve the model fit any further. However, sex was retained in all baseline models for consistency.

**Table 3:** Performance of selected comorbidity measures in assessing the association between comorbidity and selected outcome measures (Victoria)

Model	LOS^a^		LOS^b^		Hospital costs^c^	
	AUC (95% CI)	Model fit AIC	McFadden’s Adjusted R^2^	Model fit AIC	Adjusted R^2^	Model fit AIC
(i) Baseline model	0.748 (0.745–0.750)	145,468	0.080	579,331	0.312	304,799
(ii) Baseline model + selected comorbidities (individually modelled with binary representation)	0.760 (0.757–0.763)	142,523	0.088	572,274	0.359	297,352
(iii) Baseline model + selected comorbidities (modelled as a weighted summed score)^3^	0.760 (0.757–0.762)	142,582	0.088	572,673	0.357	297,674
(iv) Baseline model + comorbidity using CCI weights	0.752 (0.749–0.754)	144,554	0.083	577,080	0.328	302,263
(v) Baseline model + comorbidity using Quan weights	0.752 (0.749–0.754)	144,527	0.082	577,610	0.325	302,663
(vi) Baseline model + ECM	0.761 (0.759–0.764)	142,174	0.089	571,537	0.366	296,207
(vii) Baseline model + 23 comorbidities common to LOS and cost outcomes (binary representation)/Baseline model + 8 comorbidities common to readmission outcomes (binary representation)	0.760 (0.757–0.762)	142,603	0.088	448,263	0.358	297,494
Model	All-cause 30-day readmission^d^		Non-planned 30-day readmission^e^			
	AUC (95% CI)	Model fit AIC	AUC (95% CI)		Model fit AIC	
(i) Baseline model	0.611 (0.606–0.615)	99,275	0.623 (0.618–0.628)		75,455	
(ii) Baseline model + selected comorbidities (individually modelled with binary representation)	0.625 (0.621–0.630)	98,445	0.637 (0.632–0.642)		74,950	
(iii) Baseline model + selected comorbidities (modelled as a weighted summed score)^f^	0.625 (0.620–0.629)	98,473	0.636 (0.631–0.642)		74,946	
(iv) Baseline model + comorbidity using CCI weights	0.622 (0.617–0.626)	98,662	0.633 (0.628–0.638)		75,096	
(v) Baseline model + comorbidity using Quan weights	0.620 (0.615–0.624)	98,824	0.631 (0.626–0.637)		75,174	
(vi) Baseline model + ECM	0.626 (0.622–0.631)	98,457	0.639 (0.634–0.644)		74,912	
(vii) Baseline model + 23 comorbidities common to LOS and cost outcomes (binary representation)/Baseline model + 8 comorbidities common to readmission outcomes (binary representation)	0.622 (0.617–0.626)	98,707	0.635 (0.630–0.640)		75,010	

Notes

^a^ Baseline model includes age, sex, injury severity, injury type, body region, geographic region, SEIFA deciles and country of birth; outcome = overnight stay discharge (logistic model)

^b^ Baseline model includes age, sex, injury severity, injury type, body region, SEIFA deciles and country of birth; outcome = LOS of non-same day discharges with 1–30 days stay (negative binomial model)

^c^ Baseline model includes age, sex, injury severity, injury type, body region, SEIFA deciles, geographic region (metropolitan and regional) and country of birth; outcome = hospital costs (Ln transformed linear model)

^d^ Baseline model includes age, sex, injury type, body region, geographic region and country of birth; outcome = all cause 30-day readmission (logistic model)

^e^ Baseline model includes age, sex, injury severity, injury type, body region, geographic region and SEIFA deciles; outcome = non-planned 30 day readmission (logistic model)

^f^ See Methods for weight calculation, excludes weights resulting from an OR < 1.2

See Table 4 for selected comorbidities for each outcome

Patients from regional areas were more likely to stay overnight compared to patients from metropolitan areas. The baseline model for costs was similar to overnight stay which is expected given costs are highly correlated to LOS. Region of residence was associated with readmissions.

#### Fitting comorbidity using various measures

Models *ii-vii* are baseline models with comorbidity fitted using the newly derived binary (*ii*) and weighted comorbidity indices (*iii*), existing indices (CCI (*iv*), updated CCI (*v*) and ECM (*vi*)) and the parsimonious indices (conditions common to burden/readmission outcomes alone) (*vii*) (Table 3). Results from fitting comorbidity using *other* forms are also presented in an appendix (Appendix Table A2). The model coefficients and weights for each comorbidity, by outcome are presented in Table 4. ROC curves, prediction plots and residual plots of the various models are presented in Appendices A1.1, A1.2,A1.3, A1.4, A1.5.

**Table 4:** Risk adjusted odds ratios, incident rate ratios, beta-coefficients and suggested weights

		Australian Injury Comorbidity Indices				Existing comorbidity indices			
		Overnight stay		LOS		CCI	Updated CCI per Quan et al. (2011)	ECM point scores per van Walraven et al. (2009)	
		OR (95% CI)	Injury comorbidity index weight	IRR (95% CI)	Injury comorbidity index weight				
15–24 years		0.43 (0.41–0.45)		0.41 (0.40–0.42)					
25–44 years		0.48 (0.46–0.50)		0.45 (0.45–0.46)					
45–64 years		0.63 (0.60–0.66)		0.57 (0.56–0.58)					
65–84 years		Reference group		Reference group					
85+ years		1.16 (1.09–1.22)		1.26 (1.24–1.29)					
Female sex		1.11 (1.08–1.14)		1.12 (1.11–1.13)					
Serious injury		4.92 (4.56–5.32)		1.83 (1.80–1.86)					
HIV/AIDS		–	–	–	–	6	4	0	
Alcohol dependence		1.38 (1.29–1.48)	1	1.17 (1.14–1.20)*	–	#	#	0	
Drug dependence		2.34 (2.01–2.72)	2	1.56 (1.49–1.64)	2	#	#	-7	
Any malignancy		7.39 (4.52–12.08)	7	1.56 (1.40–1.73)	2	2	2	4	
Blood loss anemia		5.98 (1.84–19.37)	6	1.40 (1.20–1.63)	1	#	#	−2	
Cardiac arrhythmias		2.45 (2.15–2.80)	2	1.32 (1.28–1.36)	1	#	#	5	
Cerebrovascular disease		–	–	1.18 (1.08–1.29)*	–	1	0	##	
Chronic pulmonary disease		6.29 (4.26–9.30)	6	1.46 (1.38–1.54)	1	1	1	3	
Coagulopathy		3.74 (2.80–4.99)	4	1.43 (1.35–1.51)	1	#	#	3	
Congestive heart failure		7.06 (4.27–11.67)	7	1.27 (1.20–1.34)	1	1	2	7	
Deficiency anemias		8.70 (4.73–16.00)	9	1.62 (1.50–1.76)	2	#	#	−2	
Dementia		2.00 (1.70–2.34)	2	–	–	1	2	##	
Depression		3.25 (2.89–3.65)	3	1.93 (1.86–2.01)	2	#	#	−3	
Diabetes with chronic complications		1.42 (1.26–1.60)	1	1.27 (1.23–1.31)	1	2	1	0	
Diabetes without complications		1.20 (1.13–1.28)	1	1.12 (1.09–1.14)	–	1	0	0	
Hemiplegia/paraplegia		5.03 (3.12–8.10)	5	1.62 (1.47–1.78)	2	2	2	7	
Hypertension complicated		–	–	–	–	#	#	0	
Hypertension uncomplicated		2.49 (2.15–2.88)	2	1.25 (1.21–1.29)	1	#	#	0	
Hypothyroidism		21.25 (2.92–154.54)	21	1.72 (1.49–2.00)	2	#	#	0	
Metastatic solid tumor		–	–	1.32 (1.15–1.51)	1	6	6	12	
Mild liver disease		1.63 (1.38–1.93)	2	1.27 (1.20–1.34)	1	1	2	11	
Moderate or severe liver disease		20.78 (2.85–151.33)	21	1.83 (1.53–2.19)	2	3	4	11	
Myocardial infarction		–	–	–	–	1	0	##	
Obesity		4.41 (2.27–8.56)	4	2.10 (1.85–2.39)	2	#	#	−4	
Peptic ulcer disease		–	–	1.55 (1.25–1.91)	2	1	0	0	
Peripheral vascular disease		3.11 (2.35–4.13)	3	1.88 (1.76–2.02)	2	1	0	2	
Psychoses		5.54 (3.96–7.74)	6	3.14 (2.91–3.40)	3	#	#	0	
Pulmonary circulation disorders		–	–	1.81 (1.52–2.15)	2	#	#	4	
Renal disease including renal failure		1.58 (1.32–1.89)	2	1.17 (1.12–1.21)*	–	2	1	5	
Rheumatic disease including some other connective tissue disorders		33.10 (4.59–238.47)	33	1.93 (1.67–2.24)	2	1	1	0	
Valvular disease		5.28 (2.30–12.13)	5	1.36 (1.22–1.52)	1	#	#	−1	
	Australian Injury Comorbidity Indices						Existing comorbidity indices		
	Hospital cost		All-cause 30-day readmission		Non-planned 30-day readmission		CCI	Updated CCI per Quan et al. (2011)	ECM point scores per van Walraven et al. (2009)
	Beta coefficient (95% CI)	Injury comorbidity index weight^1^	OR (95% CI)	Injury comorbidity index weight	OR (95% CI)	Injury comorbidity index weight			
15–24 years	−0.25 (−0.28--0.23)		0.47 (0.44–0.50)		0.51 (0.47–0.55)				
25–44 years	− 0.21 (− 0.23--0.19)		0.58 (0.55–0.61)		0.64 (0.60–0.68)				
45–64 years	−0.16 (− 0.18--0.14)		0.70 (0.67–0.74)		0.69 (0.65–0.73)				
65–84 years	Reference group		Reference group		Reference group				
85+ years	0.02 (0.00–0.04)		1.01 (0.96–1.06)		1.18 (1.12–1.26)				
Female sex	−0.03 (−0.04--0.01)		0.97 (0.94–1.01)		0.95 (0.91–0.99)				
serious injury	0.89 (0.86–0.91)		–		1.07 (1.00–1.14)				
HIV/AIDS	–	–	–	–	–	–	6	4	0
Alcohol dependence	0.20 (0.17–0.23)	1	1.23 (1.14–1.33)	1	1.35 (1.24–1.47)	1	#	#	0
Drug dependence	0.47 (0.41–0.52)	2	–	–	1.46 (1.23–1.72)	1	#	#	−7
Any malignancy	0.74 (0.65–0.83)	2	4.10 (3.45–4.86)	4	–	–	2	2	4
Blood loss anemia	0.61 (0.45–0.78)	2	–	–	–	–	#	#	−2
Cardiac arrhythmias	0.48 (0.44–0.52)	2	–	–	–	–	#	#	5
Cerebrovascular disease	–	–	–	–	–	–	1	0	##
Chronic pulmonary disease	0.57 (0.51–0.64)	2	1.45 (1.26–1.67)	1	1.63 (1.40–1.90)	2	1	1	3
Coagulopathy	0.49 (0.43–0.56)	2	1.48 (1.27–1.72)	1	1.60 (1.36–1.88)	2	#	#	3
Congestive heart failure	0.38 (0.31–0.45)	1	–	–	1.40 (1.19–1.65)	1	1	2	7
Deficiency anemias	0.66 (0.56–0.75)	2	–	–	–	–	#	#	−2
Dementia	0.16 (0.11–0.20)*	–	–	–	–	–	1	2	##
Depression	0.63 (0.59–0.67)	2	1.38 (1.24–1.53)	1	–	–	#	#	−3
Diabetes with chronic complications	0.26 (0.22–0.30)	1	1.26 (1.15–1.38)	1	1.33 (1.20–1.47)	1	2	1	0
Diabetes without complications	0.10 (0.07–0.12)*	–	1.18 (1.11–1.26)*	–	1.22 (1.14–1.31)	1	1	0	0
Hemiplegia/paraplegia	0.70 (0.60–0.79)	2	–	–	–	–	2	2	7
Hypertension complicated	0.48 (0.20–0.76)	2	–	–	–	–	#	#	0
Hypertension uncomplicated	0.42 (0.38–0.46)	2	–	–	–	–	#	#	0
Hypothyroidism	0.56 (0.39–0.74)	2	–	–	–	–	#	#	0
Metastatic solid tumor	–	–	–	–	2.42 (1.83–3.21)	2	6	6	12
Mild liver disease	0.32 (0.25–0.38)	1	1.74 (1.50–2.03)	2	1.89 (1.60–2.24)	2	1	2	11
Moderate or severe liver disease	0.81 (0.60–1.01)	2	–	–	–	–	3	4	11
Myocardial infarction	0.24 (0.10–0.38)	1	–	–	–	–	1	0	##
Obesity	0.80 (0.65–0.95)	2	–	–	–	–	#	#	−4
Peptic ulcer disease	0.65 (0.40–0.91)	2	–	–	–	–	1	0	0
Peripheral vascular disease	0.75 (0.66–0.83)	2	–	–	–	–	1	0	2
Psychoses	1.04 (0.95–1.13)	3	1.69 (1.36–2.08)	2	1.57 (1.23–2.00)	2	#	#	0
Pulmonary circulation disorders	0.79 (0.58–0.99)	2	–	–	–	–	#	#	4
Renal disease including renal failure	0.19 (0.14–0.24)	1	2.02 (1.83–2.21)	2	1.67 (1.50–1.86)	2	2	1	5
Rheumatic disease including some other connective tissue disorders	0.83 (0.66–1.01)	2	–	–	–	–	1	1	0
Valvular disease	0.41 (0.28–0.55)	2	–	–	–	–	#	#	−1

Note:

- Condition not significantly associated with outcome

# Not included in CCI list

## Not included in ECM list

*Excluded from weighted index as OR < 1.2

1. Weight = exp. (beta)

#### Interaction effects

Interaction effects between age and sex, comorbidities and age, and comorbidities and sex, were also modelled. The age-sex interaction was selected because of their known interactions with disease [33], while the age and sex with comorbidity interactions were selected due to the expectation that the severity and impact of comorbidity can vary with age and sex. The interaction terms improved model fit but provided very little or no improvement to the predictive ability of the models, and were therefore dropped from the analysis. Plots representing interaction effects are included in Appendix A2.

#### Overnight stay

The Australian Injury Comorbidity Index for overnight stay (AICI-os) with 24 comorbidities performed better than the CCI, performed equally to the ECM with the added advantage that it uses fewer comorbidities than the ECM.

The best fit model, as indicated by the lowest AICs; was model *vi* (containing the ECM), followed by model *ii* (containing the AICI-os). Models containing the CCI (*iv*) and updated CCI (*v*) had much poorer fit. Comparing predictive abilities using the AUC and false negative rates (Table 5), the AICI-os and the ECM performed best; a false negative rate in this instance is when a patient with overnight stay is incorrectly classified as discharged on the same day. Details of model performance can be found in Appendix A3. Discrimination analysis results can be found in Appendix Table A3.

**Table 5:** False negative rates for various indices by outcome in population groups

Outcome	Number of patients in initial cohort	Number (%) of patients with outcome	Number (%) of patients classified as not having the outcome when likely to have using the;			
			outcome-specific binary index	parsimonious index	CCI	ECM
	Adults (> = 15 years) - all injuries					
At least one overnight stay	140,094	99,275 (70.9)	30,093/99273 (30.3)	30,059/99273 (30.3)	30,868/99273 (31.1)	30,008/99219 (30.2)
All-cause 30-day readmission	136,670	16,672 (12.2)	6833/16672 (41.0)	6803/16672 (40.8)	6828/16672 (41.0)	6790/16672 (40.7)
Non-planned 30-day readmission	136,670	11,150 (8.2)	4440/11150 (39.8)	4449/11150 (39.9)	4527/11150 (40.6)	4437/11150 (39.8)
	Children (< 15 years) - all injuries					
At least one overnight stay	21,240	11,062 (52.1)	3537/21194 (32.1)	3537/21194 (32.1)	3829/21238 (34.6)	3827/21189 (34.8)
All-cause 30-day readmission	21,196	1274 (6.0)	486/21179 (38.2)	487/21179 (38.2)	491/21183 (38.5)	474/21141 (37.2)
Non-planned 30-day readmission	21,196	804 (3.8)	303/21046 (37.7)	303/21056 (37.7)	303/21101 (37.7)	343/21006 (42.7)
	Patients 65 years and above					
At least one overnight stay	49,820	41,600 (83.5)	11,092/49525 (26.9)	11,163/49525 (27.0)	11,806/49820 (28.4)	11,097/49480 (26.9)
All-cause 30-day readmission	47,968	7948 (16.6)	3589/47968 (45.2)	3546/47968 (44.6)	3609/47968 (45.4)	3484 /47961 (43.8)
Non-planned 30-day readmission	47,968	5424 (11.3)	2378/47968 (43.8)	2373/47968 (43.8)	2381/47968 (43.9)	2287/47961 (42.2)
	Adult males					
At least one overnight stay	76,211	51,046 (67.0)	16,312/76109 (32.0)	16,336/76109 (32.1)	16,670/76210 (32.7)	16,334/76034 (32.1)
All-cause 30-day readmission	74,206	8359 (11.3)	3280/74205 (39.2)	3304/74205 (39.5)	3272/74205 (39.1)	3262/74205 (39.0)
Non-planned 30-day readmission	74,206	5626 (7.6)	2157/74205 (38.3)	2133/74205 (37.9)	2143/74205 (38.1)	2088/74205 (37.1)
	Adult females					
At least one overnight stay	63,883	48,229 (75.5)	14,205/63702 (29.6)	14,187/63702 (29.5)	14,594/63882 (30.3)	14,138/63675 (29.4)
All-cause 30-day readmission	62,464	8313 (13.3)	3623/62463 (43.6)	3602/62463 (43.3)	3588/62463 (43.2)	3570/62459 (42.9)
Non-planned 30-day readmission	62,464	5524 (8.8)	2351/62463 (42.6)	2292/62463 (41.5)	2346/62463 (42.5)	2301/62459 (41.7)
	Adults with non-severe injuries					
At least one overnight stay	119,916	79,942 (66.7)	26,684/119915 (33.4)	26,885/119915 (33.6)	27,455/119915 (34.3)	26,672/119889 (33.4)
All-cause 30-day readmission^a^						
Non-planned 30-day readmission	117,951	9030 (7.7)	3560/117950 (39.4)	3553/117950 (39.4)	3639/117950 (40.3)	3570/117950 (39.5)
	Adults with intracranial injury^b^					
At least one overnight stay	5456	4093 (75.0)	883/5190 (23.1)	886/5190 (23.2)	915/5456 (22.4)	882/5136 (23.4)
All-cause 30-day readmission	5026	633 (12.6)	235/5026 (37.1)	237/5026 (37.4)	236/5026 (37.3)	231/5026 (36.5)
Non-planned 30-day readmission	5026	492 (9.8)	184/5026 (37.4)	180/5026 (36.6)	181/5026 (36.8)	188/5018 (38.2)
	Patients with hip fractures 45 years and above^c^					
At least one overnight stay	161,334	110,337 (68.4)	36,536/161332 (33.1)	36,714/161332 (33.3)	37,331/161332 (33.8)	36,497/161277 (33.1)
All-cause 30-day readmission	7400	1115 (15.1)	452/7400 (40.5)	519/7400 (46.6)	491/7400 (44.0)	464/7394 (41.6)
Non-planned 30-day readmission	7400	818 (11.1)	382/7400 (46.7)	393/7400 (48.0)	306/7400 (37.4)	344/7394 (42.1)
	Adults with blunt trauma^d^					
At least one overnight stay	94,698	71,004 (75.0)	19,844/94629 (28.0)	19,924/94629 (28.1)	20,331/94696 (28.6)	19,973/94545 (28.2)
All-cause 30-day readmission	92,217	12,169 (13.2)	5174/92204 (42.5)	5210/92204 (42.8)	5201/92204 (42.7)	5069/ 92,204 (41.7)
Non-planned 30-day readmission	92,217	8313 (9.0)	3441/92204 (41.4)	3423/92204 (41.2)	3451/92204 (41.5)	3380/92177 (40.7)
	Adults with penetrating trauma^e^					
At least one overnight stay	13,313	7937 (59.6)	3165/13262 (40.1)	3161/13262 (40.1)	3245/13313 (40.9)	3143/13256 (39.9)
All-cause 30-day readmission	13,075	1058 (8.1)	460/13071 (43.5)	466/13071 (44.1)	461/13071 (43.6)	456/13045 (43.2)
Non-planned 30-day readmission	13,075	651 (5.0)	270/13058 (41.5)	270/13059 (41.5)	291/13059 (44.7)	275/13017 (42.2)
	NSW validation (age > =15 years)					
At least one overnight stay	201,791	146,177 (72.4)	46,323/146176 (31.7)	46,336/146176 (31.7)	46,670/146176 (31.9)	46,102/146176 (31.5)
All-cause 30-day readmission	193,522	28,237 (14.6)	11,911/28237 (42.2)	11,815/28237 (41.8)	11,956/28237 (42.3)	11,999/28237 (42.5)
Non-planned 30-day readmission	193,522	15,988 (8.3)	6209/15988 (38.8)	6269/15988 (39.2)	6321/15988 (39.5)	6308/15988 (39.5)
	WA validation (age > =15 years)					
At least one overnight stay	71,771	51,296 (71.5)	17,114/51296 (33.4)	17,181/51296 (33.5)	17,409/51296 (33.9)	17,072/51296 (33.3)
All-cause 30-day readmission	69,164	9132 (13.2)	3592/9132 (39.3)	3662/9132 (40.1)	3647/ 9132 (39.9)	3623/9132 (39.7)
Non-planned 30-day readmission	69,164	5684 (8.2)	2093/5684 (36.8)	2098/5684 (36.9)	2162/5684 (38.0)	2081/5684 (36.6)

Notes

Cut-off for classification tables set to maximise sensitivity and specificity

^a^. Injury severity was not found to be associated with all-cause 30-day readmission, therefore no validation was carried out in this subgroup

^b^. Intracranial injury = ICD-10-AM codes S06.00 - S06.9

^c^. Hip fractures = ICD-10 codes S72.0 - S72.2

^d^. Blunt trauma = ICD-10 codes V00-V99, W00-W19, W20-W24, W30-W31, W50-W52, X50, X79-X82, Y00-Y05 and Y29-Y32

^e^. Penetrating trauma = ICD-10 codes W53, W54, W55, W57, W58, W59, W25, W26, W27, W28, W29, W45, W32, W34, X72-X74, X78, X93-X95, X99, Y22-Y24 andY28

#### LOS (overnight stay patients)

Based on model fit, the new index (the Australian Injury Comorbidity Index for LOS (AICI-los)) fits better than the CCI. The AICI-los does not fit as well as the ECM, the only trade-off lies in modelling thirty conditions in the ECM as opposed to only 27 in the AICI-los. In terms of predictive abilities, the differences between models were relatively small. Details of model performance can be found in Appendix A3.

#### Cost

Similar to the AICI-los, the Australian Injury Comorbidity Index for costs (AICI-cost) with 28 comorbidities fits better than the CCI and less so than the ECM. Once again, the trade-off between the AICI-cost and the ECM is the number of conditions.

The best fit was once again seen in models with the ECM, followed by the AICI-cost while the CCI and updated CCI had a poorer fit. Predictive power in terms of the adjusted R^2^ was best in the model with the ECM (36.6%), followed by AICI-cost (35.9%), while predictive powers of the CCI (32.8%) and updated CCI (32.5%) were lower.

#### All-cause 30-day readmission

The Australian Injury Comorbidity Index for all-cause 30-day readmission (AICI-acr) with 10 comorbidities exhibits similar capacity in terms of model fit and predictive power as the existing indices except that it includes fewer conditions than the CCI and ECM.

In terms of model fit, the best were models with the ECM, followed by the AICI-acr, while once again the CCI and updated CCI had a poorer fit. The AUC for all models for this outcome was close to but less than 0.7, indicating that the power of the models was somewhat poor and could probably be improved by factors not already adjusted for. Further details can be found in Appendix A3.

#### Non-planned 30-day readmissions

The best fit was again observed for models with the ECM, followed by the Australian Injury Comorbidity Index for non-planed 30-day readmissions (AICI-npr) with 11 comorbidities and poorer fit with the CCI and updated CCI. There were no significant differences between all the models in terms of AUC statistics and false negative rates. Similar to all-cause readmissions, the AICI-npr has the advantage of having fewer conditions than the CCI and ECM.

#### Parsimonious indices

A binary index (model *vii*) (Table 3) with 23 conditions common in the LOS and cost indices (Australian Injury Comorbidity Index for burden (AICI-b)) and one with eight conditions common to readmissions (Australian Injury Comorbidity Index for readmissions (AICI-r)) was also derived (see Appendix Table A4 for conditions included). Overall, comparing the AUCs, false negative rates and R^2^s showed that the two parsimonious indices (AICI-b and AICI-r) can be used for predicting the corresponding outcomes without much loss in predictive capacities (Table 3 and Table 5).

### Comparison of conditions included in new and existing indices

Table 4 shows the corresponding weights for each comorbidity by outcome for the AICIs, CCI, Quan update to CCI and van Walraven update to ECM weights. Conditions like HIV/AIDS, cerebrovascular disease, dementia, metastatic solid tumors, myocardial infarction and pulmonary circulation disorders are allocated weights in the existing indices, while for some of the outcomes they show no association in the new indices.

### Internal and external validations

The newly derived indices along with the CCI and ECM were validated in subgroups of the Victorian population. A detailed discussion of results can be found in Appendix A4 while model results are presented in Appendix Table A5 and false negative rates in Table 5. The new indices and the ECM validated better than the CCI in general across most subgroups for burden outcomes. For readmission outcomes, there was no difference in predictive powers across new and existing indices, the only difference being that the ECM had significantly lower false negative rates among older adults, females and blunt trauma patients.

External validations were carried out on the entire adult portion of the interstate cohorts. Comorbidity prevalence for these are presented in Appendix Table A6. An explanation of the validation analysis can be found in Appendix A4 with model results in Appendix Table A7 and false negative rates in Table 5. For overnight stay, all indices validated well; NSW a little better than WA and the new indices and ECM better than the CCI, but no significant differences in false negative rates. For LOS, WA had higher R^2^s than Victoria and NSW, and the ECM performed best followed by the AICI and the CCI. For readmissions, AUCs were low (poor predictive power) for all indices and not significantly different, nor were the false negative rates. WA data overall had better predictive power than the other two states.

Suggestions on how the indices can be used are provided in Appendix A5. A summary table of conditions included in the AICIs, CCI and ECM are presented in Appendix Table A4.

## Discussion

### Main findings

The number and type of comorbidities associated with outcomes vary based on the outcome. The AICIs provide up-to-date, injury and outcome specific parsimonious indices that perform equally well to the long-listed ECM, and in certain instances outperform the widely used CCI.

### Study strengths

This study shows that pre-existing comorbidities associated with burden and readmission outcomes for injury patients are different for each outcome, enforcing the need for outcome-specific indices. For instance, conditions associated with LOS and costs were somewhat similar (though not identical) while conditions associated with readmissions were similar but far fewer in number than burden outcomes. The CCI and ECM has been cited extensively (CCI 29,383 citations and ECM 5450 citations according to Google Scholar as of October 2019) but are often applied to injury populations and non-mortality outcomes when they were originally derived for predicting mortality [34].

The fact that the association between comorbidities and outcomes vary and that the CCI performed better for predicting mortality than LOS and readmissions has been shown in other studies [14, 35]. Both a previous study (Fernando et al., 2020) [24] and the present study gave the same findings for the CCI, ECM and the AICIs.

The new indices allow a more appropriate quantification of the effect of each comorbidity on the outcomes as opposed to using existing indices that are based on older data. More specifically; conditions like HIV/AIDS and metastasis contain the highest risk scores according to the CCI, but the results from this study shows that these conditions have much smaller or no effect on burden and readmission outcomes. Myocardial infarction allocates a certain element of risk in the CCI, but according to the findings here, it only impacted the prediction of costs. Peptic ulcer disease is also allocated a risk element in the CCI but shows association only with LOS and cost and has no association with readmissions in the present study.

A study by Moor et al. (2008) further showed that the CCI weights assigned to conditions did not correspond to their study coefficients when assessing mortality for trauma patients [36] which the present study also confirmed. Moor et al. (2008) recommended three steps to creating appropriate empirical weights; 1) use a large representative database, 2) use appropriate generalizable trauma populations and 3) be up-to-date, all of which the present study has encompassed.

A number of studies in the past have indicated the need for study-specific comorbidity indices or weights [35, 37–40]. This study has shown the validity of those recommendations by deriving new indices and comparing their performance and parsimoniousness against the existing general indices. The characteristics of the populations, hospital facilities and comorbidity prevalence drive the outcomes.

The only study that derived a comorbidity index for injury populations was by Thompson et al. (2010) [41] which derived the Mortality Risk Score for Trauma using six comorbidities. The predictive power of their index was identical to the CCI and did not show any improvement whereas the indices derived in this study performed a little better.

Another strength of this study is the extensive validations of the new and existing indices carried out in subgroups and external populations. It was seen that in most instances the ECM performed best followed by the AICIs and CCI in predicting outcomes. However, the trade-off on the number of comorbidities modelled and the relevance to the outcome could be the deciding point for whether the ECM or AICI is used.

The new indices may be more versatile for use than the Multipurpose Australian Comorbidity Scoring System (MACSS) which was derived by Holman et al. (2005) [35]. The MACSS was not considered for evaluation in this study because it includes: (i) 102 comorbidities (far less parsimonious than all other indices), (ii) conditions like tuberculosis and (iii) certain symptoms and late effects which are not chronic diseases.

Moor et.al. (2008) [36] and Toson et al. (2015) [14] both claimed that a binary representation of comorbidities was sufficient for establishing the association between comorbidities and outcomes for injury patients and this study has validated that claim. Farley et al. (2005) [6] found that costs were better predicted by a count of comorbidities over the CCI; this study found that the binary representation had more predictive power than the count of comorbidities. The difference could be due to the fact that they used diagnosis clusters which we have not investigated in this study.

### Study limitations

#### Data limitations

The < 0.7 AUC statistics for assessing readmissions indicate that additional variables are required to explain these outcomes. Readmissions could be due to injuries, comorbidities, complications and health service delivery. Adjusting for the cause of readmission, which was not available in these datasets, may help improve the baseline model.

LOS and costs could also be largely driven by hospital facilities as well as the medical and surgical procedures carried out. The distance from a patient’s residence to the hospital could be another important factor, all of which was not part of the data included in this study. LOS can also be dependent on the total patient turnout at a hospital; hospitals with low resources may tend to discharge patients sooner resulting in lower LOS, likewise they could result in higher numbers of readmissions. Accounting for these in the baseline models may improve predictive abilities, but not necessarily the comorbidity indices.

#### Comorbidity capture

The capture of comorbidities in this study compare well with a previous study of injury patients that used administrative data in Australia. The proportion of *injury* patients with comorbidities in this study (for Victoria) was 19.5% while previous research for New South Wales, Queensland and South Australia [42] found 15.6%; the difference is likely to be attributable to the fact that this study included 31 conditions as opposed to the 17 CCI conditions used in the other study. However, these proportions could be low compared to other studies as the datasets used are administrative data which are not clinically rich as other registries to capture comorbidity.

##### Drawback of using administrative data

The administrative data used in this study does not capture the severity of comorbidities. It may also not capture all comorbidities as the function of most administrative datasets is for informing hospital reimbursement, therefore commodities that are not actively treated within the episode may not be indicated.

##### Lookback periods

The inclusion of lookback periods could improve comorbidity capture. However, the new indices are meant for use at the point of hospital admission, and data for lookback periods are generally unavailable at that point; therefore, inclusion of these will be useful only during research and not in clinical settings.

#### Practical application

Care should be taken when using the new indices in populations where the injury profile or the prevalence of comorbidity are different, as was seen in the subgroup analysis (Appendix A4). Further, they should probably not be used to compare the performance of specific hospitals as the driving factors of hospital performance likely vary largely on type, size and location of hospitals.

#### Case selection bias

Cases selected for 30-day readmissions analysis in the NSW and WA datasets excludes those that died within 30 days. The exclusion of these patients facing a ‘competing risk’ for readmission could incorporate some selection bias as it excludes some serious patients who may have the possibility of a readmission within 30 days. However, this proportion is generally around 1%. This exclusion was not carried out for Victoria as the hospital data in this instance was not linked with mortality data.

### Implications

Epidemiological research and resource use predictions for hospital admitted injury patients will benefit from using the AICIs that have been specifically derived and validated for this group of patients. For example, they can be used by hospitals for planning beds, and by health service administrators when budgeting for future hospital expenditure for injury patients with comorbidities.

Another advantage of such indices is that they are less resource intensive given they use information available at point of hospital admission. Clinicians can estimate LOS and the possibility of readmissions for injury patients, adjusting for the effect of comorbidities, and plan the services required accordingly. These indices do not replace clinical knowledge when deciding hospital logistics required for treating patients but maybe used in assisting with the decision-making processes.

### Future research

The AICIs can be further validated in other countries to understand if additional adjustments are required to make them more robust. Another step forward for these indices will be to incorporate comorbidity severity measures, which may improve the indices’ abilities.

## Conclusion

Comorbidities associated with burden and readmission outcomes vary with the outcome and the method used to measure the outcome. The up-to-date, injury- and outcome-specific AICIs for burden and readmissions, which are similar to the binary index such as the ECM but with fewer conditions, are sufficient for predicting outcomes and does not warrant weighted indices such as the CCI. The AICIs could be further improved by adding more information on comorbidities.

## Supplementary Information

**Additional file 1: Table S1.** Socio Economic Index for Areas (SEIFA) and country of birth details for the Victorian, NSW and WA study populations. **Table S2.** Performance of selected model fitting strategies in assessing the effect of comorbidity on selected outcome measures (Victoria). **Table S3.** Performance of new vs existing comorbidity indices using classification tables (Victoria). **Table S4.** Conditions included in the injury comorbidity indices for burden, readmissions, CCI and ECM. **Table S5.** Performance of new comorbidity indices vs existing comorbidity indices in injury sub-groups (Victoria). **Table S6.** Presence of comorbidity with mean LOS and proportion of patients with readmission outcomes in the NSW and WA study populations (> = 15 years). **Table S7.** Performance of selected model fitting strategies in assessing the effect of comorbidity on selected outcome measures (NSW and WA).

**Additional file 2: Appendix A1.1.** ROC curves for overnight stay (age > = 15 years).

**Additional file 3:** **Appendix A1.2.** Plots of predicted length of stay (days) vs observed (at least 1 overnight and LOS < =30).

**Additional file 4: Appendix A1.3.** Residual plots for costs.

**Additional file 5: Appendix A1.4.** ROC curves for all-cause 30-day readmissions (age > = 15 years).

**Additional file 6: Appendix A1.5.** ROC curves for non-planned 30-day readmissions (age > = 15 years).

**Additional file 7: Appendix A2.** Interaction plots.

**Additional file 8:** **Appendix A3.** Model performance details.

**Additional file 9:** **Appendix A4.** Internal and external validation details.

**Additional file 10: Appendix A5.** Using the Australian Injury Comorbidity Indices.

## Data Availability

The data that support the findings of this study are available from CVDL, CHeReL and DLB in WA but restrictions apply to the availability of these data, which were used under license for the current study, and so are not publicly available. The authors are not in a position to release the data (in accordance with the agreement with CVDL, CHeReL and DLB in WA).

## Abbreviations

APDC: Admitted Patient Data Collection
AIC: Akaike Information Criterion
AUC: Area Under The Curve
AUD: Australian Dollars
AICI: Australian Injury Comorbidity Index
AICI-acr: Australian Injury Comorbidity Index for all-cause 30-day readmission
AICI-b: Australian Injury Comorbidity Index for burden
AICI-cost: Australian Injury Comorbidity Index for costs
AICI-los: Australian Injury Comorbidity Index for LOS
AICI-npr: Australian Injury Comorbidity Index for non-planed 30-day readmissions
AICI-os: Australian Injury Comorbidity Index for overnight stay
AICI-r: Australian Injury Comorbidity Index for readmissions
CHeReL: Centre for Health Record Linkage
CVDL: Centre for Victorian Data Linkage
CCI: Charlson Comorbidity Index
DLB: Data Linkage Branch
ECM: Elixhauser Comorbidity Measure
HMDC: Hospital Morbidity Data Collection
IRR: Incident Rate Ratio
ICD-10-AM: International Statistical Classification of Diseases and Related Health Problems, Tenth Revision, Australian Modifications
ICISS: International Statistical Classification of Diseases based Injury Severity Score
IQR: Inter Quartile Range
LOS: Length Of Stay
MACSS: Multipurpose Australian Comorbidity Scoring System
NSW: New South Wales
OR: Odds Ratio
SEIFA: Socio Economic Indexes For Areas
VAED: Victorian Admitted Episodes Dataset
WA: Western Australia
