# Supplementary material for: Australian Injury Comorbidity Indices (AICIs) to predict burden and readmission among hospital-admitted injury patients
Source: BMC Health Serv Res. 2021 Feb 15;21:149. doi: 10.1186/s12913-021-06149-1 (PMC7885207; doi:10.1186/s12913-021-06149-1)
Supplement: Supplementary file 1 — Additional file 1: Table S1. Socio Economic Index for Areas (SEIFA) and country of birth details for the Victorian, NSW and WA study populations. Table S2. Performance of selected model fitting strategies in assessing the effect of comorbidity on selected outcome measures (Victoria). Table S3. Performance of new vs existing comorbidity indices using classification tables (Victoria). Table S4. Conditions included in the injury comorbidity indices for burden, readmissions, CCI and ECM. Table S5. Performance of new comorbidity indices vs existing comorbidity indices in injury sub-groups (Victoria). Table S6. Presence of comorbidity with mean LOS and proportion of patients with readmission outcomes in the NSW and WA study populations (> = 15 years). Table S7. Performance of selected model fitting strategies in assessing the effect of comorbidity on selected outcome measures (NSW and WA). [file 12913_2021_6149_MOESM1_ESM.docx]

Table A1: Socio Economic Index for Areas (SEIFA) and country of birth details for the Victorian, NSW and WA study populations

|  | Patients admitted^1^ between July 2012 and June 2014, n (%) | | | | | | | | | | | |
| --- | --- | --- | --- | --- | --- | --- | --- | --- | --- | --- | --- | --- |
|  | Victoria | | | | NSW | | | | WA | | | |
|  | n | % | At least one comorbidity (%) | Count of comorbidities, mean (95% CI) | n | % | At least one comorbidity (%) | Count of comorbidities, mean (95% CI) | n | % | At least one comorbidity (%) | Count of comorbidities, mean (95% CI) |
| Total patients | 161334 | | | | 233521 | | | | 84877 | | | |
| **SEIFA state decile^1^** |  |  |  |  |  |  |  |  |  |  |  |  |
| 1 | 17503 | 10.8 | 21.6 | 0.31 (0.30-0.32) | 17879 | 7.7 | 20.1 | 0.29 (0.28-0.30) | 1800 | 2.1 | 36.2 | 0.46 (0.43-0.49) |
| 2 | ** | ** | 20.3 | 0.28 (0.27-0.30) | 11249 | 4.8 | 19.7 | 0.29 (0.27-0.30) | 2359 | 2.8 | 25.5 | 0.33 (0.31-0.36) |
| 3 | 10595 | 6.5 | 22.0 | 0.32 (0.31-0.33) | 12527 | 5.4 | 22.4 | 0.32 (0.31-0.34) | 1813 | 2.1 | 22.0 | 0.29 (0.26-0.31) |
| 4 | 17975 | 11.1 | 21.1 | 0.29 (0.28-0.30) | 21142 | 9.1 | 21.6 | 0.31 (0.30-0.32) | 5713 | 6.7 | 22.7 | 0.31 (0.29-0.33) |
| 5 | 10601 | 6.6 | 17.6 | 0.25 (0.24-0.26) | 20382 | 8.7 | 18.3 | 0.26 (0.25-0.27) | 5656 | 6.7 | 21.4 | 0.29 (0.28-0.31) |
| 6 | 11830 | 7.3 | 18.8 | 0.27 (0.25-0.28) | 30030 | 12.9 | 18.7 | 0.27 (0.26-0.28) | 4759 | 5.6 | 20.5 | 0.29 (0.27-0.31) |
| 7 | 11678 | 7.3 | 20.0 | 0.28 (0.27-0.29) | 24255 | 10.4 | 18.9 | 0.27 (0.26-0.28) | 10226 | 12.0 | 18.2 | 0.25 (0.24-0.26) |
| 8 | 25726 | 16.0 | 18.9 | 0.26 (0.26-0.27) | 24339 | 10.4 | 17.6 | 0.25 (0.24-0.26) | 10719 | 12.6 | 20.8 | 0.29 (0.28-0.30) |
| 9 | 23873 | 14.8 | 19.6 | 0.27 (0.27-0.28) | 31664 | 13.6 | 19.2 | 0.28 (0.28-0.29) | 24734 | 29.1 | 19.3 | 0.26 (0.25-0.27) |
| 10 | 22902 | 14.2 | 18.7 | 0.26 (0.25-0.27) | 38996 | 16.7 | 16.9 | 0.25 (0.24-0.25) | 16777 | 19.8 | 19.2 | 0.26 (0.25-0.27) |
| 999 | * | * | * | * | 1058 | 0.5 | 33.9 | 0.45 (0.40-0.49) | 321 | 0.4 | 34.6 | 0.42 (0.35-0.50) |
|  |  |  |  |  |  |  |  |  |  |  |  |  |
| **Country of birth** |  |  |  |  |  |  |  |  |  |  |  |  |
| Australia | 121134 | 75.4 | 17.5 | 0.24 (0.24-0.24) | 178833 | 76.6 | 17.6 | 0.25 (0.25-0.25) | 60205 | 70.9 | 19.7 | 0.26 (0.26-0.27) |
| New Zealand | 2397 | 1.5 | 13.8 | 0.18 (0.16-0.20) | 4126 | 1.8 | 16.5 | 0.23 (0.21-0.25) | 3173 | 3.7 | 15.5 | 0.19 (0.17-0.21) |
| England | 4912 | 3.0 | 27.8 | 0.40 (0.38-0.42) | 8043 | 3.4 | 24.6 | 0.36 (0.34-0.37) | 6185 | 7.3 | 25.1 | 0.37 (0.35-0.39) |
| Italy | 3474 | 2.0 | 42.7 | 0.68 (0.65-0.71) | 2689 | 1.2 | 39.3 | 0.64 (0.60-0.67) | 1056 | 1.2 | 39.6 | 0.62 (0.56-0.68) |
| Greece | 2162 | 1.3 | 40.0 | 0.62 (0.58-0.66) | 1642 | 0.7 | 40.6 | 0.67 (0.62-0.72) | 147 | 0.2 | 44.9 | 0.72 (0.55-0.89) |
| India | 1657 | 1.1 | 16.4 | 0.22 (0.19-0.24) | 1607 | 0.7 | 15.6 | 0.22 (0.19-0.25) | 699 | 0.8 | 26.9 | 0.41 (0.35-0.48) |
| All other | 25598 | 15.8 | 25.0 | 0.36 (0.35-0.37) | 36581 | 15.7 | 22.7 | 0.34 (0.34-0.35) | 13412 | 15.8 | 20.4 | 0.29 (0.27-0.30) |

Notes:

1. Based on SLAs for Victoria and NSW, and LGAs for WA

Table A2: Performance of selected model fitting strategies in assessing the effect of comorbidity on selected outcome measures (Victoria)

| Model | Outcome | | | | | |
| --- | --- | --- | --- | --- | --- | --- |
|  | LOS^1.1^ | | LOS^1.2^ | | Hospital costs ^2^ | |
|  | AUC (95% CI) | Model fit AIC | McFadden's  Adjusted R^2^ | Model fit AIC | Adjusted R^2^ | Model fit AIC |
| (viii) Baseline model + presence of at least one comorbidity | 0.755 (0.752-0.757) | 143989 | 0.085 | 575238 | 0.337 | 300857 |
| (ix) Baseline model + count of comorbidities | 0.756 (0.754-0.759) | 143533 | 0.085 | 574825 | 0.345 | 299579 |
| (x) Baseline model + individual comorbidity (all 31 conditions) | 0.760 (0.757-0.763) | 142501 | 0.088 | 572265 | 0.359 | 297336 |
| (xi) Baseline model + selected comorbidities (modelled as a weighted summed score)^3^ | 0.760 (0.757-0.762) | 142570 | 0.088 | 572770 | 0.356 | 297734 |
|  |  |  |  |  |  |  |
| Baseline model deconstructed |  |  |  |  |  |  |
| Age | 0.636 (0.633-0.639) | 161731 | 0.048 | 608531 | 0.057 | 337677 |
| Age + Sex | 0.639 (0.636-0.642) | 161619 | 0.048 | 607965 | 0.058 | 337593 |
| Age + Sex + Body region | 0.701 (0.698-0.704) | 154187 | 0.059 | 597100 | 0.128 | 329418 |
| Age + Sex + Body region + Injury type | 0.733 (0.730-0.735) | 149014 | 0.074 | 584880 | 0.254 | 313180 |
| Age + Sex + Body region + Injury type + Injury severity | 0.744 (0.741-0.746) | 146299 | 0.080 | 579421 | 0.309 | 305191 |
| Age + Sex + Body region + Injury type + SEIFA |  |  |  |  |  |  |
| Age + Sex + Body region + Injury type + SEIFA + Geographic region |  |  |  |  |  |  |
| Age + Sex + Body region + Injury type + SEIFA + Geographic region + Country of birth |  |  |  |  |  |  |
| Age + Sex + Body region + Injury type + Injury severity + SEIFA | 0.745 (0.743-0.748) | 145954 | 0.080 | 579397 | 0.311 | 304940 |
| Age + Sex + Body region + Injury type + Injury severity + SEIFA + Country of birth |  |  | 0.080 | 579331 |  |  |
| Age + Sex + Body region + Injury type + Injury severity + SEIFA + Geographic region | 0.748 (0.745-0.750) | 145494 |  |  | 0.311 | 304824 |
| Age + Sex + Body region + Injury type + Injury severity + SEIFA + Geographic region + Country of birth | 0.748 (0.745-0.750) | 145468 |  |  | 0.312 | 304799 |

Notes:

1.1 Baseline model includes age, sex, injury severity, injury type, body region, geographic region, SEIFA deciles and country of birth; outcome =overnight

stay discharge (logistic model)

1.2 Baseline model includes age, sex, injury severity, injury type, body region, SEIFA deciles and country of birth; outcome = LOS of non-same day discharges

with 1-30 days stay (negative binomial model)

2. Baseline model includes age, sex, injury severity, injury type, body region, SEIFA deciles, geographic region (metropolitan and regional) and country of birth;

outcome= hospital costs (Ln transformed linear model)

3. Actual ORs used as weights, excludes weights resulting from an OR<1.2

Table A2 continued…

| Model | Outcome | | | |
| --- | --- | --- | --- | --- |
|  | All-cause 30-day readmission^1^ | | Non-planned 30-day readmission^2^ | |
|  | AUC (95% CI) | Model fit AIC | AUC (95% CI) | Model fit AIC |
| (viii) Baseline model + presence of at least one comorbidity | 0.621 (0.616-0.625) | 98920 | 0.633 (0.628-0.639) | 75142 |
| (ix) Baseline model + count of comorbidities | 0.623 (0.618-0.627) | 98776 | 0.635 (0.630-0.640) | 75039 |
| (x) Baseline model + individual comorbidity (all 31 conditions) | 0.627 (0.622-0.631) | 98427 | 0.639 (0.633-0.644) | 74924 |
| (xi) Baseline model + selected comorbidities (modelled as a weighted summed score)^3^ | 0.625 (0.620-0.629) | 98488 | 0.637 (0.631-0.642) | 74953 |
|  |  |  |  |  |
| Baseline model deconstructed |  |  |  |  |
| Age | 0.587 (0.582-0.591) | 99907 | 0.588 (0.582-0.593) | 76206 |
| Age + Sex | 0.587 (0.583-0.592) | 99909 | 0.588 (0.583-0.594) | 76206 |
| Age + Sex + Body region | 0.602 (0.598-0.607) | 99558 | 0.610 (0.605-0.615) | 75799 |
| Age + Sex + Body region + Injury type | 0.608 (0.603-0.612) | 99342 | 0.617 (0.611-0.622) | 75614 |
| Age + Sex + Body region + Injury type + Injury severity |  |  | 0.618 (0.612-0.623) | 75601 |
| Age + Sex + Body region + Injury type + SEIFA | 0.608 (0.604-0.613) | 99345 |  |  |
| Age + Sex + Body region + Injury type + SEIFA + Geographic region | 0.610 (0.606-0.614) | 99286 |  |  |
| Age + Sex + Body region + Injury type + SEIFA + Geographic region + Country of birth | 0.611 (0.606-0.615) | 99275 |  |  |
| Age + Sex + Body region + Injury type + Injury severity + SEIFA |  |  | 0.620 (0.614-0.625) | 75565 |
| Age + Sex + Body region + Injury type + Injury severity + SEIFA + Country of birth |  |  |  |  |
| Age + Sex + Body region + Injury type + Injury severity + SEIFA + Geographic region |  |  | 0.623 (0.618-0.628) | 75455 |
| Age + Sex + Body region + Injury type + Injury severity + SEIFA + Geographic region + Country of birth |  |  |  |  |

Notes:

1. Baseline model includes age, sex, injury type, body region, geographic region and country of birth; outcome=all cause 30-day readmission (logistic

model)

2. Baseline model includes age, sex, injury severity, injury type, body region, geographic region and SEIFA deciles; outcome=non-planned 30 day

readmission (logistic model)

3. Actual ORs used as weights, excludes weights resulting from an OR<1.2

Table A3: Performance of new vs existing comorbidity indices using classification tables (Victoria)

| Model | At least one overnight stay | | | | All-cause 30-day readmission | | | | Non-planned 30-day readmission | | | |
| --- | --- | --- | --- | --- | --- | --- | --- | --- | --- | --- | --- | --- |
| Baseline | Classified | TRUE D | ~D | Total | Classified | TRUE D | ~D | Total | Classified | TRUE D | ~D | Total |
|  |  |  |  |  |  |  |  |  |  |  |  |  |
|  |  | 68031 | 12828 | 80859 |  | 12711 | 72482 | 85193 |  | 6571 | 51251 | 57822 |
|  |  | 31242 | 27991 | 59233 |  | 3961 | 47514 | 51475 |  | 4579 | 74267 | 78846 |
|  |  |  |  |  |  |  |  |  |  |  |  |  |
|  | Total | 99273 | 40819 | 140092 | Total | 16672 | 119996 | 136668 | Total | 11150 | 125518 | 136668 |
|  |  |  |  |  |  |  |  |  |  |  |  |  |
|  | Classified + if predicted Pr(D) | >= .66 |  |  | Classified + if predicted Pr(D) | >= .1 |  |  | Classified + if predicted Pr(D) | >= .083 |  |  |
|  | True D defined as newsameday!= 0 |  |  |  | True D defined as event != 0 | |  |  | True D defined as event != 0 |  |  |  |
|  |  |  |  |  |  |  |  |  |  |  |  |  |
|  | Sensitivity | Pr( + D) | 68.53% |  | Sensitivity | Pr( + D) | 76.24% |  | Sensitivity | Pr( + D) | 58.93% |  |
|  | Specificity | Pr( -~D) | 68.57% |  | Specificity | Pr( -~D) | 39.60% |  | Specificity | Pr( -~D) | 59.17% |  |
|  | Positive predictive value | Pr( D +) | 84.14% |  | Positive predictive value | Pr( D +) | 14.92% |  | Positive predictive value | Pr( D +) | 11.36% |  |
|  | Negative predictive value | Pr(~D -) | 47.26% |  | Negative predictive value | Pr(~D -) | 92.31% |  | Negative predictive value | Pr(~D -) | 94.19% |  |
|  |  |  |  |  |  |  |  |  |  |  |  |  |
|  | False + rate for true ~D | Pr( +~D) | 31.43% |  | False + rate for true ~D | Pr( +~D) | 60.40% |  | False + rate for true ~D | Pr( +~D) | 40.83% |  |
|  | False - rate for true D | Pr( - D) | 31.47% |  | False - rate for true D | Pr( - D) | 23.76% |  | False - rate for true D | Pr( - D) | 41.07% |  |
|  | False + rate for classified + | Pr(~D +) | 15.86% |  | False + rate for classified + | Pr(~D +) | 85.08% |  | False + rate for classified + | Pr(~D +) | 88.64% |  |
|  | False - rate for classified - | Pr( D -) | 52.74% |  | False - rate for classified - | Pr( D -) | 7.69% |  | False - rate for classified - | Pr( D -) | 5.81% |  |
|  |  |  |  |  |  |  |  |  |  |  |  |  |
|  | Correctly classified |  | 68.54% |  | Correctly classified |  |  | 44.07% | Correctly classified |  | 59.15% |  |
| Baseline + CCI | Classified | TRUE D | ~D | Total | Classified | TRUE D | ~D | Total | Classified | TRUE D | ~D | Total |
|  |  |  |  |  |  |  |  |  |  |  |  |  |
|  |  | 68405 | 12654 | 81059 |  | 9844 | 49257 | 59101 |  | 6623 | 50275 | 56898 |
|  |  | 30868 | 28165 | 59033 |  | 6828 | 70739 | 77567 |  | 4527 | 75243 | 79770 |
|  |  |  |  |  |  |  |  |  |  |  |  |  |
|  | Total | 99273 | 40819 | 140092 | Total | 16672 | 119996 | 136668 | Total | 11150 | 125518 | 136668 |
|  |  |  |  |  |  |  |  |  |  |  |  |  |
|  | Classified + if predicted Pr(D) | >= .658 |  |  | Classified + if predicted Pr(D) | >= .121 |  |  | Classified + if predicted Pr(D) | >= .081 |  |  |
|  | True D defined as newsameday!= 0 |  |  |  | True D defined as event != 0 | |  |  | True D defined as event != 0 |  |  |  |
|  |  |  |  |  |  |  |  |  |  |  |  |  |
|  | Sensitivity | Pr( + D) | 68.91% |  | Sensitivity | Pr( + D) | 59.05% |  | Sensitivity | Pr( + D) | 59.40% |  |
|  | Specificity | Pr( -~D) | 69.00% |  | Specificity | Pr( -~D) | 58.95% |  | Specificity | Pr( -~D) | 59.95% |  |
|  | Positive predictive value | Pr( D +) | 84.39% |  | Positive predictive value | Pr( D +) | 16.66% |  | Positive predictive value | Pr( D +) | 11.64% |  |
|  | Negative predictive value | Pr(~D -) | 47.71% |  | Negative predictive value | Pr(~D -) | 91.20% |  | Negative predictive value | Pr(~D -) | 94.32% |  |
|  |  |  |  |  |  |  |  |  |  |  |  |  |
|  | False + rate for true ~D | Pr( +~D) | 31.00% |  | False + rate for true ~D | Pr( +~D) | 41.05% |  | False + rate for true ~D | Pr( +~D) | 40.05% |  |
|  | False - rate for true D | Pr( - D) | 31.09% |  | False - rate for true D | Pr( - D) | 40.95% |  | False - rate for true D | Pr( - D) | 40.60% |  |
|  | False + rate for classified + | Pr(~D +) | 15.61% |  | False + rate for classified + | Pr(~D +) | 83.34% |  | False + rate for classified + | Pr(~D +) | 88.36% |  |
|  | False - rate for classified - | Pr( D -) | 52.29% |  | False - rate for classified - | Pr( D -) | 8.80% |  | False - rate for classified - | Pr( D -) | 5.68% |  |
|  |  |  |  |  |  |  |  |  |  |  |  |  |
|  | Correctly classified |  | 68.93% |  | Correctly classified |  | 58.96% |  | Correctly classified |  | 59.90% |  |
| Baseline + updated CCI by Quan et al. (2011) | Classified | TRUE D | ~D | Total | Classified | TRUE D | ~D | Total | Classified | TRUE D | ~D | Total |
|  |  |  |  |  |  |  |  |  |  |  |  |  |
|  |  | 68557 | 12723 | 81280 |  | 9831 | 49183 | 59014 |  | 6699 | 51272 | 57971 |
|  |  | 30716 | 28096 | 58812 |  | 6841 | 70813 | 77654 |  | 4451 | 74246 | 78697 |
|  |  |  |  |  |  |  |  |  |  |  |  |  |
|  | Total | 99273 | 40819 | 140092 | Total | 16672 | 119996 | 136668 | Total | 11150 | 125518 | 136668 |
|  |  |  |  |  |  |  |  |  |  |  |  |  |
|  | Classified + if predicted Pr(D) | >= .656 |  |  | Classified + if predicted Pr(D) | >= .122 |  |  | Classified + if predicted Pr(D) | >= .081 |  |  |
|  | True D defined as newsameday!= 0 |  |  |  | True D defined as event != 0 | |  |  | True D defined as event != 0 |  |  |  |
|  |  |  |  |  |  |  |  |  |  |  |  |  |
|  | Sensitivity | Pr( + D) | 69.06% |  | Sensitivity | Pr( + D) | 58.97% |  | Sensitivity | Pr( + D) | 60.08% |  |
|  | Specificity | Pr( -~D) | 68.83% |  | Specificity | Pr( -~D) | 59.01% |  | Specificity | Pr( -~D) | 59.15% |  |
|  | Positive predictive value | Pr( D +) | 84.35% |  | Positive predictive value | Pr( D +) | 16.66% |  | Positive predictive value | Pr( D +) | 11.56% |  |
|  | Negative predictive value | Pr(~D -) | 47.77% |  | Negative predictive value | Pr(~D -) | 91.19% |  | Negative predictive value | Pr(~D -) | 94.34% |  |
|  |  |  |  |  |  |  |  |  |  |  |  |  |
|  | False + rate for true ~D | Pr( +~D) | 31.17% |  | False + rate for true ~D | Pr( +~D) | 40.99% |  | False + rate for true ~D | Pr( +~D) | 40.85% |  |
|  | False - rate for true D | Pr( - D) | 30.94% |  | False - rate for true D | Pr( - D) | 41.03% |  | False - rate for true D | Pr( - D) | 39.92% |  |
|  | False + rate for classified + | Pr(~D +) | 15.65% |  | False + rate for classified + | Pr(~D +) | 83.34% |  | False + rate for classified + | Pr(~D +) | 88.44% |  |
|  | False - rate for classified - | Pr( D -) | 52.23% |  | False - rate for classified - | Pr( D -) | 8.81% |  | False - rate for classified - | Pr( D -) | 5.66% |  |
|  |  |  |  |  |  |  |  |  |  |  |  |  |
|  | Correctly classified |  | 68.99% |  | Correctly classified |  | 59.01% |  | Correctly classified |  | 59.23% |  |
|  |  |  |  |  |  |  |  |  |  |  |  |  |
| Baseline + ECM | Classified | TRUE D | ~D | Total | Classified | TRUE D | ~D | Total | Classified | D | ~D | Total |
|  |  |  |  |  |  |  |  |  |  |  |  |  |
|  |  | 69211 | 12370 | 81581 |  | 9882 | 49021 | 58903 |  | 6713 | 50131 | 56844 |
|  |  | 30008 | 28449 | 58457 |  | 6790 | 70975 | 77765 |  | 4437 | 75387 | 79824 |
|  |  |  |  |  |  |  |  |  |  |  |  |  |
|  | Total | 99219 | 40819 | 140038 | Total | 16672 | 119996 | 136668 | Total | 11150 | 125518 | 136668 |
|  |  |  |  |  |  |  |  |  |  |  |  |  |
|  | Classified + if predicted Pr(D) | >= .651 |  |  | Classified + if predicted Pr(D) | >= .12 |  |  | Classified + if predicted Pr(D) | >= .08 |  |  |
|  | True D defined as newsameday!= 0 |  |  |  | True D defined as event != 0 | |  |  | True D defined as event != 0 |  |  |  |
|  |  |  |  |  |  |  |  |  |  |  |  |  |
|  | Sensitivity | Pr( + D) | 69.76% |  | Sensitivity | Pr( + D) | 59.27% |  | Sensitivity | Pr( + D) | 60.21% |  |
|  | Specificity | Pr( -~D) | 69.70% |  | Specificity | Pr( -~D) | 59.15% |  | Specificity | Pr( -~D) | 60.06% |  |
|  | Positive predictive value | Pr( D +) | 84.84% |  | Positive predictive value | Pr( D +) | 16.78% |  | Positive predictive value | Pr( D +) | 11.81% |  |
|  | Negative predictive value | Pr(~D -) | 48.67% |  | Negative predictive value | Pr(~D -) | 91.27% |  | Negative predictive value | Pr(~D -) | 94.44% |  |
|  |  |  |  |  |  |  |  |  |  |  |  |  |
|  | False + rate for true ~D | Pr( +~D) | 30.30% |  | False + rate for true ~D | Pr( +~D) | 40.85% |  | False + rate for true ~D | Pr( +~D) | 39.94% |  |
|  | False - rate for true D | Pr( - D) | 30.24% |  | False - rate for true D | Pr( - D) | 40.73% |  | False - rate for true D | Pr( - D) | 39.79% |  |
|  | False + rate for classified + | Pr(~D +) | 15.16% |  | False + rate for classified + | Pr(~D +) | 83.22% |  | False + rate for classified + | Pr(~D +) | 88.19% |  |
|  | False - rate for classified - | Pr( D -) | 51.33% |  | False - rate for classified - | Pr( D -) | 8.73% |  | False - rate for classified - | Pr( D -) | 5.56% |  |
|  |  |  |  |  |  |  |  |  |  |  |  |  |
|  | Correctly classified |  | 69.74% |  | Correctly classified |  | 59.16% |  | Correctly classified |  | 60.07% |  |
| Baseline + binary injury comorbidity index | Classified | TRUE D | ~D | Total | Classified | TRUE D | ~D | Total | Classified | TRUE D | ~D | Total |
|  |  |  |  |  |  |  |  |  |  |  |  |  |
|  |  | 69180 | 12377 | 81557 |  | 9839 | 48937 | 58776 |  | 6710 | 50539 | 57249 |
|  |  | 30093 | 28442 | 58535 |  | 6833 | 71059 | 77892 |  | 4440 | 74979 | 79419 |
|  |  |  |  |  |  |  |  |  |  |  |  |  |
|  | Total | 99273 | 40819 | 140092 | Total | 16672 | 119996 | 136668 | Total | 11150 | 125518 | 136668 |
|  |  |  |  |  |  |  |  |  |  |  |  |  |
|  |  |  |  |  |  |  |  |  | Classified + if predicted Pr(D) | >= .08 |  |  |
|  | Classified + if predicted Pr(D) | >= .652 |  |  | Classified + if predicted Pr(D) | >= .12 |  |  | True D defined as event != 0 |  |  |  |
|  | True D defined as newsameday!= 0 |  |  |  | True D defined as event != 0 | |  |  |  |  |  |  |
|  |  |  |  |  |  |  |  |  | Sensitivity | Pr( + D) | 60.18% |  |
|  | Sensitivity | Pr( + D) | 69.69% |  | Sensitivity | Pr( + D) | 59.02% |  | Specificity | Pr( -~D) | 59.74% |  |
|  | Specificity | Pr( -~D) | 69.68% |  | Specificity | Pr( -~D) | 59.22% |  | Positive predictive value | Pr( D +) | 11.72% |  |
|  | Positive predictive value | Pr( D +) | 84.82% |  | Positive predictive value | Pr( D +) | 16.74% |  | Negative predictive value | Pr(~D -) | 94.41% |  |
|  | Negative predictive value | Pr(~D -) | 48.59% |  | Negative predictive value | Pr(~D -) | 91.23% |  |  |  |  |  |
|  |  |  |  |  |  |  |  |  | False + rate for true ~D | Pr( +~D) | 40.26% |  |
|  | False + rate for true ~D | Pr( +~D) | 30.32% |  | False + rate for true ~D | Pr( +~D) | 40.78% |  | False - rate for true D | Pr( - D) | 39.82% |  |
|  | False - rate for true D | Pr( - D) | 30.31% |  | False - rate for true D | Pr( - D) | 40.98% |  | False + rate for classified + | Pr(~D +) | 88.28% |  |
|  | False + rate for classified + | Pr(~D +) | 15.18% |  | False + rate for classified + | Pr(~D +) | 83.26% |  | False - rate for classified - | Pr( D -) | 5.59% |  |
|  | False - rate for classified - | Pr( D -) | 51.41% |  | False - rate for classified - | Pr( D -) | 8.77% |  |  |  |  |  |
|  |  |  |  |  |  |  |  |  | Correctly classified |  | 59.77% |  |
|  | Correctly classified |  | 69.68% |  | Correctly classified |  | 59.19% |  |  |  |  |  |
| Baseline + weighted injury comorbidity index | Classified | TRUE D | ~D | Total | Classified | TRUE D | ~D | Total | Classified | TRUE D | ~D | Total |
|  |  |  |  |  |  |  |  |  |  |  |  |  |
|  |  | 69138 | 12396 | 81534 |  | 9854 | 49149 | 59003 |  | 6704 | 50686 | 57390 |
|  |  | 30135 | 28423 | 58558 |  | 6818 | 70847 | 77665 |  | 4446 | 74832 | 79278 |
|  |  |  |  |  |  |  |  |  |  |  |  |  |
|  | Total | 99273 | 40819 | 140092 | Total | 16672 | 119996 | 136668 | Total | 11150 | 125518 | 136668 |
|  |  |  |  |  |  |  |  |  |  |  |  |  |
|  | Classified + if predicted Pr(D) | >= .654 |  |  | Classified + if predicted Pr(D) | >= .12 |  |  |  |  |  |  |
|  | True D defined as newsameday!= 0 |  |  |  | True D defined as event != 0 | |  |  | Classified + if predicted Pr(D) | >= .08 |  |  |
|  |  |  |  |  |  |  |  |  | True D defined as event != 0 |  |  |  |
|  | Sensitivity | Pr( + D) | 69.64% |  | Sensitivity | Pr( + D) | 59.11% |  |  |  |  |  |
|  | Specificity | Pr( -~D) | 69.63% |  | Specificity | Pr( -~D) | 59.04% |  | Sensitivity | Pr( + D) | 60.13% |  |
|  | Positive predictive value | Pr( D +) | 84.80% |  | Positive predictive value | Pr( D +) | 16.70% |  | Specificity | Pr( -~D) | 59.62% |  |
|  | Negative predictive value | Pr(~D -) | 48.54% |  | Negative predictive value | Pr(~D -) | 91.22% |  | Positive predictive value | Pr( D +) | 11.68% |  |
|  |  |  |  |  |  |  |  |  | Negative predictive value | Pr(~D -) | 94.39% |  |
|  | False + rate for true ~D | Pr( +~D) | 30.37% |  | False + rate for true ~D | Pr( +~D) | 40.96% |  |  |  |  |  |
|  | False - rate for true D | Pr( - D) | 30.36% |  | False - rate for true D | Pr( - D) | 40.89% |  | False + rate for true ~D | Pr( +~D) | 40.38% |  |
|  | False + rate for classified + | Pr(~D +) | 15.20% |  | False + rate for classified + | Pr(~D +) | 83.30% |  | False - rate for true D | Pr( - D) | 39.87% |  |
|  | False - rate for classified - | Pr( D -) | 51.46% |  | False - rate for classified - | Pr( D -) | 8.78% |  | False + rate for classified + | Pr(~D +) | 88.32% |  |
|  |  |  |  |  |  |  |  |  | False - rate for classified - | Pr( D -) | 5.61% |  |
|  | Correctly classified |  | 69.64% |  | Correctly classified |  | 59.05% |  |  |  |  |  |
|  |  |  |  |  |  |  |  |  | Correctly classified |  | 59.66% |  |
| Baseline + parsimonious injury comorbidity index | Classified | TRUE D | ~D | Total | Classified | TRUE D | ~D | Total | Classified | TRUE D | ~D | Total |
|  |  |  |  |  |  |  |  |  |  |  |  |  |
|  |  | 69214 | 12426 | 81640 |  | 9869 | 49399 | 59268 |  | 6701 | 50826 | 57527 |
|  |  | 30059 | 28393 | 58452 |  | 6803 | 70597 | 77400 |  | 4449 | 74692 | 79141 |
|  |  |  |  |  |  |  |  |  |  |  |  |  |
|  | Total | 99273 | 40819 | 140092 | Total | 16672 | 119996 | 136668 | Total | 11150 | 125518 | 136668 |
|  |  |  |  |  |  |  |  |  |  |  |  |  |
|  | Classified + if predicted Pr(D) | >= .651 |  |  | Classified + if predicted Pr(D) | >= .121 |  |  | Classified + if predicted Pr(D) | >= .08 |  |  |
|  | True D defined as newsameday!= 0 |  |  |  | True D defined as event != 0 | |  |  | True D defined as event != 0 |  |  |  |
|  |  |  |  |  |  |  |  |  |  |  |  |  |
|  | Sensitivity | Pr( + D) | 69.72% |  | Sensitivity | Pr( + D) | 59.20% |  | Sensitivity | Pr( + D) | 60.10% |  |
|  | Specificity | Pr( -~D) | 69.56% |  | Specificity | Pr( -~D) | 58.83% |  | Specificity | Pr( -~D) | 59.51% |  |
|  | Positive predictive value | Pr( D +) | 84.78% |  | Positive predictive value | Pr( D +) | 16.65% |  | Positive predictive value | Pr( D +) | 11.65% |  |
|  | Negative predictive value | Pr(~D -) | 48.57% |  | Negative predictive value | Pr(~D -) | 91.21% |  | Negative predictive value | Pr(~D -) | 94.38% |  |
|  |  |  |  |  |  |  |  |  |  |  |  |  |
|  | False + rate for true ~D | Pr( +~D) | 30.44% |  | False + rate for true ~D | Pr( +~D) | 41.17% |  | False + rate for true ~D | Pr( +~D) | 40.49% |  |
|  | False - rate for true D | Pr( - D) | 30.28% |  | False - rate for true D | Pr( - D) | 40.80% |  | False - rate for true D | Pr( - D) | 39.90% |  |
|  | False + rate for classified + | Pr(~D +) | 15.22% |  | False + rate for classified + | Pr(~D +) | 83.35% |  | False + rate for classified + | Pr(~D +) | 88.35% |  |
|  | False - rate for classified - | Pr( D -) | 51.43% |  | False - rate for classified - | Pr( D -) | 8.79% |  | False - rate for classified - | Pr( D -) | 5.62% |  |
|  |  |  |  |  |  |  |  |  |  |  |  |  |
|  | Correctly classified |  | 69.67% |  | Correctly classified |  | 58.88% |  | Correctly classified |  | 59.56% |  |
|  |  |  |  |  |  |  |  |  |  |  |  |  |

Table A4: Conditions included in the injury comorbidity indices for burden, readmissions, CCI and ECM

| Comorbidity | | Outcome | | | | | | | | | |
| --- | --- | --- | --- | --- | --- | --- | --- | --- | --- | --- | --- |
|  |  | Overnight stay  (AICI-os)^1^ | LOS  (AICI-los)^2^ | Cost  (AICI-cost)^3^ | Burden (AICI-b)^4^ | All-cause  30-day readmission  (AICI-acr)^5^ | | Non-planned  30-day readmission  (AICI-npr)^6^ | Readmissions (AICI-r)^7^ | CCI | ECM |
| HIV/AIDS | | X | X | X | X | X | | X | X | ✓ | ✓ |
| Alcohol dependence | | ✓ | ✓ | ✓ | ✓ | ✓ | | ✓ | ✓ | X | ✓ |
| Drug dependence | | ✓ | ✓ | ✓ | ✓ | X | | ✓ | X | X | ✓ |
| Any malignancy | | ✓^8^ | ✓^8^ | ✓^8^ | ✓^8^ | ✓^8^ | | X | X | ✓^8^ | ✓^9^ |
| Blood loss anaemia | | ✓ | ✓ | ✓ | ✓ | X | | X | X | X | ✓ |
| Cardiac arrhythmias | | ✓ | ✓ | ✓ | ✓ | X | | X | X | X | ✓ |
| Cerebrovascular disease | | X | ✓ | X | X | X | | X | X | ✓ | X |
| Chronic pulmonary disease | | ✓ | ✓ | ✓ | ✓ | ✓ | | ✓ | ✓ | ✓ | ✓ |
| Coagulopathy | | ✓ | ✓ | ✓ | ✓ | ✓ | | ✓ | ✓ | X | ✓ |
| Congestive heart failure | | ✓ | ✓ | ✓ | ✓ | X | | ✓ | X | ✓ | ✓ |
| Deficiency anaemias | | ✓ | ✓ | ✓ | ✓ | X | | X | X | X | ✓ |
| Dementia | | ✓ | X | ✓ | X | X | | X | X | ✓ | X |
| Depression | | ✓ | ✓ | ✓ | ✓ | ✓ | | X | X | X | ✓ |
| Diabetes with chronic complications | | ✓ | ✓ | ✓ | ✓ | ✓ | | ✓ | ✓ | ✓ | ✓ |
| Diabetes without complications | | ✓ | ✓ | ✓ | ✓ | ✓ | | ✓ | ✓ | ✓ | ✓ |
| Fluid and electrolyte disorders | | - | - | - | - | - | | - | - | - | ✓ |
| Hemiplegia/paraplegia | | ✓ | ✓ | ✓ | ✓ | X | | X | X | ✓ | ✓ |
| Hypertension complicated | | X | X | ✓ | X | X | | X | X | X | ✓ |
| Hypertension uncomplicated | | ✓ | ✓ | ✓ | ✓ | X | | X | X | X |  |
| Hypothyroidism | | ✓ | ✓ | ✓ | ✓ | X | | X | X | X | ✓ |
| Metastatic solid tumor | | X | ✓ | X | X | X | | ✓ | X | ✓ | ✓ |
| Mild liver disease | | ✓ | ✓ | ✓ | ✓ | ✓ | | ✓ | ✓ | ✓ | ✓ |
| Moderate or severe liver disease | | ✓ | ✓ | ✓ | ✓ | X | | X | X | ✓ |  |
| Myocardial infarction | | X | X | ✓ | X | X | | X | X | ✓ | X |
| Obesity | | ✓ | ✓ | ✓ | ✓ | X | | X | X | X | ✓ |
| Other neurological disorders | | - | - | - | - | - | | - | - | - | ✓ |
| Peptic ulcer disease | | X | ✓ | ✓ | X | X | | X | X | ✓ | ✓ |
| Peripheral vascular disease | | ✓ | ✓ | ✓ | ✓ | X | | X | X | ✓ | ✓ |
| Psychoses | | ✓ | ✓ | ✓ | ✓ | ✓ | | ✓ | ✓ | X | ✓ |
| Pulmonary circulation disorders | | X | ✓ | ✓ | X | X | | X | X | X | ✓ |
| Renal disease including renal failure | | ✓ | ✓ | ✓ | ✓ | ✓ | | ✓ | ✓ | ✓ | ✓ |
| Rheumatic disease including  some other connective tissue disorders | | ✓ | ✓ | ✓ | ✓ | X | | X | X | ✓ | ✓ |
| Valvular disease | | ✓ | ✓ | ✓ | ✓ | X | | X | X | X | ✓ |
| Weight loss | | - | - | - | - | - | | - | - | - | ✓ |
| Notes: | | | | | |  |  |  |  |  |  |
| 1. AICI-os - Australian Injury Comorbidity Index - overnight stay | | | | | |  |  |  |  |  |  |
| 2. AICI-los - Australian Injury Comorbidity Index - length of stay for non-same day discharges | | | | | |  |  |  |  |  |  |
| 3. AICI-cost - Australian Injury Comorbidity Index -direct hospital cost | | | | | |  |  |  |  |  |  |
| 4. AICI-b - Australian Injury Comorbidity Index - burden (includes cost, overnight stay and LOS for overnight stays) | | | | | |  |  |  |  |  |  |
| 5. AICI-acr - Australian Injury Comorbidity Index - all-cause 30-day readmission | | | | | |  |  |  |  |  |  |
| 6. AICI-npr - Australian Injury Comorbidity Index - non-planned 30-day readmission | | | | | |  |  |  |  |  |  |
| 7. AICI-r -Australian Injury Comorbidity Index - readmissions (includes all-cause and non-planned 30-day readmissions) | | | | | |  |  |  |  |  |  |
| 8. Includes lymphoma, solid tumors without metastasis and leukaemia | | | | | |  |  |  |  |  |  |
| 9. Includes lymphoma and solid tumors without metastasis | | | | | |  |  |  |  |  |  |

Table A5: Performance of new comorbidity indices vs existing comorbidity indices in injury sub-groups (Victoria)

| Model | Children (<15 years) | | Adults (>=65 years) | | Males | | Females | |
| --- | --- | --- | --- | --- | --- | --- | --- | --- |
|  | AUC (95% CI) | Model fit AIC | AUC (95% CI) | Model fit AIC | AUC (95% CI) | Model fit AIC | AUC (95% CI) | Model fit AIC |
|  | At least one overnight stay^1^ | | | | | | | |
| Baseline model | 0.721 (0.714-0.728) | 26030 | 0.777 (0.772-0.782) | 37678 | 0.730 (0.726-0.733) | 84607 | 0.764 (0.760-0.768) | 60340 |
| Baseline model + AICI-os | 0.723 (0.717-0.730) | 25933 | 0.803 (0.798-0.807) | 36104 | 0.740 (0.737-0.744) | 83177 | 0.779 (0.775-0.783) | 58809 |
| Baseline model + comorbidity using CCI weights | 0.722 (0.715-0.729) | 26013 | 0.789 (0.784-0.794) | 37021 | 0.734 (0.730-0.737) | 84115 | 0.769 (0.765-0.773) | 59904 |
| Baseline model + comorbidity using ECM | 0.724 (0.717-0.731) | 25910 | 0.805 (0.801-0.810) | 35906 | 0.741 (0.738-0.745) | 83013 | 0.780 (0.777-0.784) | 58627 |
| Baseline model + AICI-b | 0.723 (0.717-0.730) | 25933 | 0.801 (0.797-0.806) | 36193 | 0.740 (0.737-0.744) | 83196 | 0.778 (0.775-0.782) | 58870 |
|  | All-cause 30-day readmission^2^ | | | | | | | |
| Baseline model | 0.654 (0.638-0.670) | 9302 | 0.549 (0.542-0.556) | 42952 | 0.638 (0.631-0.644) | 50644 | 0.581 (0.575-0.587) | 48474 |
| Baseline model + AICI-acr | 0.660 (0.644-0.676) | 9276 | 0.577 (0.570-0.584) | 42500 | 0.649 (0.643-0.656) | 50188 | 0.599 (0.592-0.605) | 48118 |
| Baseline model + comorbidity using CCI weights | 0.656 (0.640-0.672) | 9296 | 0.572 (0.565-0.579) | 42607 | 0.646 (0.639-0.652) | 50334 | 0.595 (0.589-0.602) | 48185 |
| Baseline model + comorbidity using ECM | 0.663 (0.647-0.679) | 9258 | 0.580 (0.573-0.587) | 42527 | 0.651 (0.644-0.657) | 50200 | 0.601 (0.595-0.608) | 48132 |
| Baseline model + AICI-r | 0.656 (0.640-0.672) | 9295 | 0.570 (0.563-0.577) | 42643 | 0.647 (0.640-0.653) | 50309 | 0.595 (0.588-0.601) | 48254 |
|  | Non-planned 30-day readmission^3^ | | | | | | | |
| Baseline model | 0.663 (0.644-0.682) | 6554 | 0.566 (0.558-0.574) | 33663 | 0.651 (0.644-0.658) | 38501 | 0.595 (0.588-0.603) | 36852 |
| Baseline model + AICI-npr | 0.665 (0.646-0.684) | 6552 | 0.589 (0.581-0.597) | 33406 | 0.664 (0.656-0.671) | 38212 | 0.611 (0.603-0.618) | 36653 |
| Baseline model + comorbidity using CCI weights | 0.664 (0.645-0.683) | 6555 | 0.587 (0.579-0.595) | 33455 | 0.658 (0.651-0.665) | 38342 | 0.609 (0.601-0.617) | 36664 |
| Baseline model + comorbidity using ECM | 0.672 (0.652-0.691) | 6527 | 0.595 (0.587-0.603) | 33391 | 0.665 (0.658-0.673) | 38212 | 0.614 (0.606-0.622) | 36636 |
| Baseline model + AICI-r | 0.665 (0.645-0.684) | 6553 | 0.586 (0.578-0.594) | 33430 | 0.662 (0.655-0.669) | 38236 | 0.609 (0.601-0.616) | 36683 |
|  | LOS^4^ | | | | | | | |
|  | McFadden's  Adjusted R^2^ | Model fit AIC | McFadden's  Adjusted R^2^ | Model fit AIC | McFadden's  Adjusted R^2^ | Model fit AIC | McFadden's  Adjusted R^2^ | Model fit AIC |
| Baseline model | 0.045 | 32273 | 0.027 | 213529 | 0.079 | 219966 | 0.068 | 231716 |
| Baseline model + AICI-los | 0.053 | 32007 | 0.032 | 212380 | 0.090 | 217428 | 0.075 | 230120 |
| Baseline model + comorbidity using CCI weights | 0.047 | 32215 | 0.030 | 212970 | 0.082 | 219182 | 0.071 | 231196 |
| Baseline model + comorbidity using ECM | 0.054 | 31944 | 0.033 | 212162 | 0.091 | 217203 | 0.076 | 229915 |
| Baseline model + AICI-b | 0.053 | 32013 | 0.032 | 212407 | 0.090 | 217470 | 0.075 | 230136 |
|  | Cost^5^ | | | | | | | |
|  | Adjusted R^2^ | Model fit AIC | Adjusted R^3^ | Model fit AIC | Adjusted R^3^ | Model fit AIC | Adjusted R^4^ | Model fit AIC |
| Baseline model | 0.263 | 46417 | 0.344 | 108902 | 0.273 | 163891 | 0.349 | 140235 |
| Baseline model + AICI-cost | 0.274 | 46169 | 0.402 | 105731 | 0.320 | 159997 | 0.397 | 136675 |
| Baseline model + comorbidity using CCI weights | 0.266 | 46366 | 0.368 | 107603 | 0.289 | 162597 | 0.367 | 138982 |
| Baseline model + comorbidity using ECM | 0.277 | 46106 | 0.411 | 105183 | 0.326 | 159537 | 0.406 | 136014 |
| Baseline model + AICI-b | 0.274 | 46174 | 0.401 | 105792 | 0.319 | 160093 | 0.397 | 136723 |

Notes:

1. Baseline model includes age, sex, injury severity, injury type, body region, geographic region, SEIFA deciles and country of birth; outcome =overnight stay

discharge (logistic model)

2. Baseline model includes age, sex, injury type, body region, geographic region and country of birth; outcome=all-cause 30-day readmission (logistic model)

3. Baseline model includes age, sex, injury severity, injury type, body region, geographic region and SEIFA deciles; outcome=non-planned 30-day

readmission (logistic model)

4. Baseline model includes age, sex, injury severity, injury type, body region, SEIFA deciles and country of birth; outcome = LOS of non-same day discharges

with 1-30 days stay (negative binomial model)

5. Baseline model includes age, sex, injury severity, injury type, body region, SEIFA deciles, geographic region (metropolitan and regional) and country of birth;

outcome= hospital costs (Ln transformed linear model)

Table A5 continued…

| Model | Non-severe injury (adults) | | Intracranial injury^1^ (adults) | | Hip-fracture^2^ | |
| --- | --- | --- | --- | --- | --- | --- |
|  | AUC (95% CI) | Model fit AIC | AUC (95% CI) | Model fit AIC | AUC (95% CI) | Model fit AIC |
|  | At least one overnight stay^3^ | | | | | |
| Baseline model | 84607 | 138924 | 0.834 (0.822-0.846) | 4552 | 0.711 (0.709-0.714) | 180003 |
| Baseline model + AICI-os | 0.717 (0.714-0.720) | 136175 | 0.832 (0.820-0.844) | 4484 | 0.733 (0.730-0.735) | 175133 |
| Baseline model + comorbidity using CCI weights | 0.707 (0.704-0.710) | 138073 | 0.837 (0.826-0.849) | 4524 | 0.723 (0.720-0.725) | 177784 |
| Baseline model + comorbidity using ECM | 0.718 (0.716-0.721) | 135870 | 0.831 (0.819-0.843) | 4473 | 0.734 (0.731-0.736) | 174741 |
| Baseline model + AICI-b | 0.717 (0.714-0.720) | 136245 | 0.831 (0.819-0.843) | 4487 | 0.732 (0.729-0.734) | 175397 |
|  | All-cause 30-day readmission^4^ | | | | | |
| Baseline model |  |  | 0.649 (0.627-0.671) | 3699 | 0.576 (0.558-0.594) | 6238 |
| Baseline model + AICI-acr |  |  | 0.667 (0.644-0.689) | 3671 | 0.610 (0.592-0.629) | 6158 |
| Baseline model + comorbidity using CCI weights |  |  | 0.666 (0.643-0.688) | 3666 | 0.601 (0.582-0.619) | 6174 |
| Baseline model + comorbidity using ECM |  |  | 0.674 (0.651-0.696) | 3686 | 0.619 (0.601-0.638) | 6167 |
| Baseline model + AICI-r |  |  | 0.663 (0.640-0.685) | 3680 | 0.602 (0.584-0.620) | 6186 |
|  | Non-planned 30-day readmission^5^ | | | | | |
| Baseline model | 0.628 (0.622-0.634) | 62191 | 0.658 (0.635-0.682) | 3121 | 0.576 (0.555-0.597) | 5113 |
| Baseline model + AICI-npr | 0.642 (0.636-0.647) | 61771 | 0.673 (0.649-0.697) | 3115 | 0.601 (0.580-0.622) | 5084 |
| Baseline model + comorbidity using CCI weights | 0.638 (0.632-0.643) | 61900 | 0.672 (0.647-0.696) | 3107 | 0.594 (0.573-0.614) | 5082 |
| Baseline model + comorbidity using ECM | 0.643 (0.638-0.649) | 61766 | 0.681 (0.657-0.705) | 3129 | 0.615 (0.594-0.636) | 5090 |
| Baseline model + AICI-r | 0.640 (0.634-0.646) | 61822 | 0.670 (0.645-0.694) | 3116 | 0.598 (0.577-0.619) | 5083 |
|  | LOS^6^ | | | | | |
|  | McFadden's  Adjusted R^2^ | Model fit AIC | McFadden's  Adjusted R^2^ | Model fit AIC | McFadden's  Adjusted R^2^ | Model fit AIC |
| Baseline model | 0.066 | 355699 | 0.063 | 18655 | 0.000 | 37609 |
| Baseline model + AICI-los | 0.077 | 351537 | 0.067 | 18571 | 0.001 | 37594 |
| Baseline model + comorbidity using CCI weights | 0.070 | 354177 | 0.065 | 18615 | 0.000 | 37611 |
| Baseline model + comorbidity using ECM | 0.078 | 351051 | 0.069 | 18533 | 0.000 | 37597 |
| Baseline model + AICI-b | 0.077 | 351584 | 0.067 | 18572 | 0.001 | 37589 |
|  | Cost^7^ | | | | | |
|  | Adjusted R^4^ | Model fit AIC | Adjusted R^5^ | Model fit AIC | Adjusted R^5^ | Model fit AIC |
| Baseline model | 0.174 | 258804 | 0.428 | 14054 | 0.010 | 13634 |
| Baseline model + AICI-cost | 0.241 | 251241 | 0.464 | 13776 | 0.060 | 13363 |
| Baseline model + comorbidity using CCI weights | 0.199 | 256022 | 0.435 | 13998 | 0.016 | 13597 |
| Baseline model + comorbidity using ECM | 0.248 | 250355 | 0.472 | 13710 | 0.051 | 13421 |
| Baseline model + AICI-b | 0.238 | 251587 | 0.462 | 13786 | 0.043 | 13462 |

Notes:

1. Intracranial injury = ICD-10-AM codes S06.00 - S06.9

2. Hip fractures = ICD-10 codes S72.0 - S72.2

3. Baseline model includes age, sex, injury severity, injury type, body region, geographic region, SEIFA deciles and country of birth; outcome =overnight stay

discharge (logistic model)

4. Baseline model includes age, sex, injury type, body region, geographic region and country of birth; outcome=all-cause 30-day readmission (logistic model)

5. Baseline model includes age, sex, injury severity, injury type, body region, geographic region and SEIFA deciles; outcome=non-planned 30-day

readmission (logistic model)

6. Baseline model includes age, sex, injury severity, injury type, body region, SEIFA deciles and country of birth; outcome = LOS of non-same day discharges

with 1-30 days stay (negative binomial model)

7. Baseline model includes age, sex, injury severity, injury type, body region, SEIFA deciles, geographic region (metropolitan and regional) and country of birth;

outcome= hospital costs (Ln transformed linear model)

Table A5 continued….

| Model | Blunt trauma^1^ | | Penetrating trauma^2^ | |
| --- | --- | --- | --- | --- |
|  | AUC (95% CI) | Model fit AIC | AUC (95% CI) | Model fit AIC |
|  | At least one overnight stay^3^ | | | |
| Baseline model | 0.779 (0.775-0.782) | 88409 | 0.639 (0.630-0.649) | 17039 |
| Baseline model + AICI-os | 0.789 (0.785-0.791) | 86740 | 0.651 (0.642-0.661) | 16852 |
| Baseline model + comorbidity using CCI weights | 0.783 (0.779-0.786) | 87763 | 0.643 (0.634-0.653) | 16990 |
| Baseline model + comorbidity using ECM | 0.789 (0.786-0.792) | 86508 | 0.651 (0.642-0.660) | 16847 |
| Baseline model + AICI-b | 0.788 (0.785-0.791) | 86801 | 0.651 (0.642-0.661) | 16850 |
|  | All-cause 30-day readmission^4^ | | | |
| Baseline model | 0.592 (0.587-0.597) | 70900 | 0.576 (0.558-0.594) | 7358 |
| Baseline model + AICI-acr | 0.609 (0.603-0.614) | 70304 | 0.600 (0.581-0.618) | 7293 |
| Baseline model + comorbidity using CCI weights | 0.606 (0.601-0.611) | 70442 | 0.582 (0.564-0.600) | 7337 |
| Baseline model + comorbidity using ECM | 0.611 (0.605-0.616) | 70309 | 0.605 (0.586-0.623) | 7281 |
| Baseline model + AICI-r | 0.605 (0.600-0.611) | 70463 | 0.591 (0.573-0.609) | 7324 |
|  | Non-planned 30-day readmission^5^ | | | |
| Baseline model | 0.602 (0.596-0.608) | 54966 | 0.583 (0.560-0.606) | 5188 |
| Baseline model + AICI-npr | 0.617 (0.611-0.623) | 54609 | 0.604 (0.581-0.627) | 5158 |
| Baseline model + comorbidity using CCI weights | 0.613 (0.607-0.619) | 54713 | 0.590 (0.567-0.613) | 5172 |
| Baseline model + comorbidity using ECM | 0.621 (0.614-0.627) | 54560 | 0.614 (0.591-0.637) | 5148 |
| Baseline model + AICI-r | 0.615 (0.609-0.621) | 54640 | 0.604 (0.581-0.627) | 5156 |
|  | LOS^6^ | | | |
|  | McFadden's  Adjusted R^2^ | Model fit AIC | McFadden's  Adjusted R^2^ | Model fit AIC |
| Baseline model | 0.074 | 333109 | 0.038 | 30897 |
| Baseline model + AICI-los | 0.080 | 331008 | 0.062 | 30112 |
| Baseline model + comorbidity using CCI weights | 0.077 | 332208 | 0.041 | 30775 |
| Baseline model + comorbidity using ECM | 0.081 | 330737 | 0.063 | 30081 |
| Baseline model + AICI-b | 0.080 | 331045 | 0.062 | 30118 |
|  | Cost^7^ | | | |
|  | Adjusted R^6^ | Model fit AIC | Adjusted R^6^ | Model fit AIC |
| Baseline model | 0.355 | 216229 | 0.175 | 25933 |
| Baseline model + AICI-cost | 0.394 | 211588 | 0.209 | 25481 |
| Baseline model + comorbidity using CCI weights | 0.371 | 214309 | 0.184 | 25810 |
| Baseline model + comorbidity using ECM | 0.400 | 210863 | 0.211 | 25457 |
| Baseline model + AICI-b | 0.394 | 211660 | 0.208 | 25486 |

Notes:

1. Blunt trauma = ICD-10 codes V00-V99, W00-W19, W20-W24, W30-W31, W50-W52, X50, X79-X82, Y00-Y05, Y29-Y32 and Y85

2. Penetrating trauma = ICD-10 codes W53, W54, W55, W57,W58,W59,W25,W26,W27,W28,W29,W45,W32,X72-X74,X78,X93-X95,X99,Y22-Y24 andY28

3. Baseline model includes age, sex, injury severity, injury type, body region, geographic region, SEIFA deciles and country of birth; outcome =overnight stay

discharge (logistic model)

4. Baseline model includes age, sex, injury type, body region, geographic region and country of birth; outcome=all-cause 30-day readmission (logistic model)

5. Baseline model includes age, sex, injury severity, injury type, body region, geographic region and SEIFA deciles; outcome=non-planned 30-day

readmission (logistic model)

6. Baseline model includes age, sex, injury severity, injury type, body region, SEIFA deciles and country of birth; outcome = LOS of non-same day discharges

with 1-30 days stay (negative binomial model)

7. Baseline model includes age, sex, injury severity, injury type, body region, SEIFA deciles, geographic region (metropolitan and regional) and country of birth;

outcome= hospital costs (Ln transformed linear model)

Table A6: Presence of comorbidity with mean LOS and proportion of patients with readmission outcomes in the NSW and WA study populations (>=15 years)

| Comorbidity | NSW | | | | |
| --- | --- | --- | --- | --- | --- |
|  | Index LOS (n=201791) | | Readmissions (n=193522) | All-cause 30-day  readmission | Non-planned 30-day readmission |
|  | n (%) | Index LOS  (mean, 95% CI) | n (%) | % with condition that had the outcome | % with condition that had the outcome |
| HIV/AIDS | 49 (0.0) | 4.8 (2.6-6.9) | 43 (0.0) | 11.6 | * |
| Alcohol dependence | 10970 (5.4) | 6.2 (5.9-6.5) | 10209 (5.3) | 16.4 | 11.1 |
| Drug dependence | 3108 (1.5) | 7.2 (6.5-7.9) | 2777 (1.4) | 19.1 | 14.3 |
| Any malignancy | 846 (0.4) | 21.0 (19.4-22.6) | 595 (0.3) | 34.8 | 25.9 |
| Blood loss anemia | 152 (0.1) | 28.9 (23.6-34.3) | 137 (0.1) | 23.4 | 16.8 |
| Cardiac arrhythmias | 5588 (2.8) | 20.7 (20.0-21.5) | 5023 (2.6) | 20.6 | 14.5 |
| Cerebrovascular disease | 1078 (0.5) | 24.3 (22.4-26.2) | 964 (0.5) | 20.0 | 15.2 |
| Chronic pulmonary disease | 1676 (0.8) | 20.1 (18.9-21.3) | 1466 (0.8) | 25.3 | 19.2 |
| Coagulopathy | 1225 (0.6) | 22.6 (20.7-24.4) | 1062 (0.5) | 23.7 | 18.1 |
| Congestive heart failure | 1407 (0.7) | 28.1 (26.6-29.6) | 1100 (0.6) | 25.5 | 19.8 |
| Deficiency anemias | 683 (0.3) | 25.3 (22.8-27.7) | 637 (0.3) | 25.3 | 18.2 |
| Dementia | 5694 (2.8) | 14.4 (13.9-15.0) | 5180 (2.7) | 16.8 | 14.4 |
| Depression | 5713 (2.8) | 10.3 (9.6-10.9) | 5530 (2.9) | 18.7 | 12.0 |
| Diabetes with chronic complications | 4466 (2.2) | 19.8 (18.9-20.7) | 4089 (2.1) | 25.7 | 18.1 |
| Diabetes without complications | 10717 (5.3) | 12.2 (11.7-12.6) | 10191 (5.3) | 18.6 | 12.8 |
| Hemiplegia/paraplegia | 962 (0.5) | 27.9 (25.1-30.7) | 867 (0.4) | 20.1 | 13.8 |
| Hypertension complicated | 44 (0.0) | 23.0 (16.0-30.1) | 36 (0.0) | 25.0 | 13.9 |
| Hypertension uncomplicated | 7057 (3.5) | 21.5 (20.8-22.2) | 6447 (3.3) | 20.7 | 13.6 |
| Hypothyroidism | 312 (0.2) | 23.3 (19.9-26.6) | 295 (0.2) | 22.4 | 14.9 |
| Metastatic solid tumor | 477 (0.2) | 20.1 (18.2-22.1) | 301 (0.2) | 37.9 | 29.2 |
| Mild liver disease | 1351 (0.7) | 12.8 (11.1-14.6) | 1194 (0.6) | 23.1 | 16.3 |
| Moderate or severe liver disease | 159 (0.1) | 26.5 (19.6-33.4) | 126 (0.1) | 32.5 | 25.4 |
| Myocardial infarction | 455 (0.2) | 23.8 (21.4-26.2) | 365 (0.2) | 20.8 | 15.6 |
| Obesity | 501 (0.3) | 26.5 (22.4-30.6) | 475 (0.2) | 22.7 | 15.2 |
| Peptic ulcer disease | 105 (0.1) | 22.6 (16.3-28.9) | 91 (0.0) | 17.6 | 9.9 |
| Peripheral vascular disease | 447 (0.2) | 27.8 (24.2-31.4) | 407 (0.2) | 26.0 | 16.2 |
| Psychoses | 937 (0.5) | 26.9 (22.6-31.2) | 872 (0.5) | 22.9 | 17.7 |
| Pulmonary circulation disorders | 349 (0.2) | 35.8 (30.9-40.8) | 275 (0.1) | 25.5 | 19.3 |
| Renal disease including renal failure | 3457 (1.7) | 22.3 (21.4-23.2) | 2995 (1.5) | 29.8 | 19.4 |
| Rheumatic disease including some other connective tissue disorders | 307 (0.2) | 24.4 (21.0-27.7) | 287 (0.1) | 19.2 | 12.9 |
| Valvular disease | 551 (0.3) | 25.1 (22.8-27.4) | 484 (0.3) | 22.7 | 16.1 |

Table A6 continued…..

| Comorbidity | WA | | | | |
| --- | --- | --- | --- | --- | --- |
|  | Index LOS (n=71771) | | Readmissions (n=69164) | All-cause  30-day  readmission | Non-planned 30-day readmission |
|  | n (%) | Index LOS (mean, 95% CI) | n (%) | % with condition that had the outcome | % with condition that had the outcome |
| HIV/AIDS | 24 (0.0) | 4.8 (1.8-7.8) | 20 (0.0) | 35.0 | * |
| Alcohol dependence | 6307 (8.8) | 4.3 (4.0-4.6) | 5873 (3.0) | 16.9 | 13.2 |
| Drug dependence | 1347 (1.9) | 4.6 (4.0-5.2) | 1235 (0.6) | 18.6 | 15.9 |
| Any malignancy | 307 (0.4) | 17.1 (14.3-19.9) | 220 (0.1) | 47.3 | 24.5 |
| Blood loss anemia | 105 (0.2) | 20.6 (15.9-25.3) | 92 (0.0) | 17.4 | 13.0 |
| Cardiac arrhythmias | 1516 (2.1) | 18.3 (16.8-19.9) | 1353 (0.7) | 22.0 | 16.2 |
| Cerebrovascular disease | 352 (0.5) | 26.0 (20.9-31.0) | 320 (0.2) | 23.4 | 18.4 |
| Chronic pulmonary disease | 442 (0.6) | 17.0 (15.0-19.0) | 393 (0.2) | 25.2 | 18.8 |
| Coagulopathy | 517 (0.7) | 20.4 (17.6-23.1) | 445 (0.2) | 22.5 | 15.3 |
| Congestive heart failure | 429 (0.6) | 22.7 (20.4-25.1) | 341 (0.2) | 24.9 | 19.6 |
| Deficiency anemias | 216 (0.3) | 20.1 (16.1-24.0) | 204 (0.1) | 19.6 | 13.7 |
| Dementia | 1534 (2.1) | 15.2 (13.9-16.5) | 1335 (0.7) | 17.9 | 15.9 |
| Depression | 1266 (1.8) | 7.7 (6.6-8.9) | 1222 (0.6) | 25.6 | 16.8 |
| Diabetes with chronic complications | 1726 (2.4) | 14.9 (13.7-16.1) | 1592 (0.8) | 27.3 | 19.1 |
| Diabetes without complications | 4349 (6.1) | 9.5 (8.9-10.2) | 4162 (2.2) | 18.0 | 13.0 |
| Hemiplegia/paraplegia | 338 (0.5) | 28.1 (22.3-34.0) | 306 (0.2) | 20.9 | 14.4 |
| Hypertension complicated | 29 (0.0) | 21.4 (10.7-32.1) | 25 (0.0) | 40.0 | 28.0 |
| Hypertension uncomplicated | 2077 (2.9) | 18.8 (17.6-19.9) | 1884 (1.0) | 24.8 | 17.5 |
| Hypothyroidism | 73 (0.1) | 25.1 (18.7-31.4) | 68 (0.0) | 14.7 | 8.8 |
| Metastatic solid tumor | 157 (0.2) | 15.9 (13.4-18.4) | 101 (0.1) | 50.5 | 25.7 |
| Mild liver disease | 550 (0.8) | 9.0 (7.5-10.6) | 483 (0.2) | 19.3 | 15.3 |
| Moderate or severe liver disease | 55 (0.1) | 18.5 (10.6-26.4) | 40 (0.0) | 35.0 | 22.5 |
| Myocardial infarction | 126 (0.2) | 25.9 (20.9-30.8) | 99 (0.1) | 20.2 | 17.2 |
| Obesity | 140 (0.2) | 23.2 (17.2-29.3) | 133 (0.1) | 18.8 | 14.3 |
| Peptic ulcer disease | 55 (0.1) | 17.5 (11.2-23.7) | 50 (0.0) | 22.0 | * |
| Peripheral vascular disease | 391 (0.5) | 16.0 (13.8-18.3) | 364 (0.2) | 15.7 | 9.6 |
| Psychoses | 182 (0.3) | 15.0 (11.5-18.4) | 175 (0.1) | 35.4 | 25.7 |
| Pulmonary circulation disorders | 91 (0.1) | 34.1 (20.9-47.2) | 71 (0.0) | 23.9 | 16.9 |
| Renal disease including renal failure | 1095 (1.5) | 17.6 (16.0-19.1) | 969 (0.5) | 36.8 | 24.6 |
| Rheumatic disease including some other connective tissue disorders | 107 (0.2) | 22.7 (17.7-27.7) | 99 (0.1) | 28.3 | 16.2 |
| Valvular disease | 159 (0.2) | 21.9 (18.3-25.6) | 143 (0.1) | 18.9 | 14.0 |

Notes:

*Cell count 1-4 suppressed to protect confidentiality

Table A7: Performance of selected model fitting strategies in assessing the effect of comorbidity on selected outcome measures (NSW and WA)

| Model | LOS^1.1^ | | LOS^1.2^ | |
| --- | --- | --- | --- | --- |
|  | AUC (95% CI) | Model fit AIC | McFadden's  Adjusted R^2^ | Model fit AIC |
|  | NSW | | | |
| (i) Baseline model | 0.736 (0.734-0.739) | 208244 | 0.076 | 658947 |
| (ii) Baseline model + selected comorbidities (individually modelled with binary representation) | 0.748 (0.746-0.751) | 204746 | 0.084 | 653296 |
| (iii) Baseline model + selected comorbidities (modelled as a weighted summed score)^3^ | 0.747 (0.745-0.750) | 205238 | 0.084 | 653595 |
| (iv) Baseline model + comorbidity using CCI weights | 0.740 (0.737-0.742) | 207318 | 0.079 | 657108 |
| (v) Baseline model + comorbidity using Quan weights | 0.739 (0.736-0.741) | 207568 | 0.078 | 657511 |
| (vi) Baseline model + ECM | 0.750 (0.748-0.752) | 204129 | 0.086 | 652310 |
| (vii) Baseline model + 23 comorbidities common to LOS and cost outcomes (binary representation)/Baseline model + 8 comorbidities common to readmission outcomes (binary representation) | 0.748 (0.746-0.751) | 204744 | 0.084 | 653340 |
|  |  |  |  |  |
|  | WA | | | |
| (i) Baseline model | 0.718 (0.714-0.722) | 76512 | 0.093 | 226149 |
| (v) Baseline model + individual comorbidity (selected) (binary representation) | 0.731 (0.728-0.735) | 75272 | 0.102 | 223901 |
| (vi) Baseline model + comorbidity using ICI (integer value of actual weight) | 0.729 (0.725-0.733) | 75563 | 0.102 | 224038 |
| (vii) Baseline model + comorbidity using CCI weights | 0.723 (0.719-0.727) | 76035 | 0.096 | 225330 |
| (viii) Baseline model + comorbidity using Quan weights | 0.722 (0.719-0.726) | 76115 | 0.095 | 225536 |
| (ix) Baseline model + ECM | 0.734 (0.730-0.737) | 75048 | 0.103 | 223586 |
| (vii) Baseline model + 23 comorbidities common to LOS and cost outcomes (binary representation)/Baseline model + 8 comorbidities common to readmission outcomes (binary representation) | 0.731 (0.727-0.735) | 75281 | 0.102 | 223932 |

Note:

1.1 Baseline model includes age, sex, injury severity, injury type, body region, geographic region, SEIFA deciles and country of birth; outcome =overnight

stay discharge (logistic model)

1.2 Baseline model includes age, sex, injury severity, injury type, body region, SEIFA deciles and country of birth; outcome = LOS of non-same day discharges

with 1-30 days stay (negative binomial model)

Table A7 continued….

| Model | All-cause 30-day readmission^1^ | | Non-planned 30-day readmission^2^ | |
| --- | --- | --- | --- | --- |
|  | AUC (95% CI) | Model fit AIC | AUC (95% CI) | Model fit AIC |
|  | NSW | | | |
| (i) Baseline model | 0.594 (0.590-0.597) | 158291 | 0.629 (0.625-0.633) | 107407 |
| (ii) Baseline model + selected comorbidities (individually modelled with binary representation) | 0.604 (0.600-0.607) | 157706 | 0.641 (0.636-0.645) | 106765 |
| (iii) Baseline model + selected comorbidities (modelled as a weighted summed score) | 0.603 (0.599-0.606) | 157729 | 0.640 (0.636-0.645) | 106795 |
| (iv) Baseline model + comorbidity using CCI weights | 0.600 (0.597-0.604) | 157906 | 0.637 (0.633-0.642) | 106966 |
| (v) Baseline model + comorbidity using Quan weights | 0.598 (0.595-0.602) | 158057 | 0.636 (0.631-0.640) | 107052 |
| (vi) Baseline model + ECM | 0.605 (0.602-0.608) | 157666 | 0.643 (0.639-0.647) | 106704 |
| (vii) Baseline model + 23 comorbidities common to LOS and cost outcomes (binary representation)/Baseline model + 8 comorbidities common to readmission outcomes (binary representation) | 0.602 (0.598-0.605) | 157830 | 0.639 (0.634-0.643) | 106891 |
|  |  |  |  |  |
|  | WA | | | |
| (i) Baseline model | 0.625 (0.619-0.631) | 52550 | 0.656 (0.648-0.663) | 37822 |
| (v) Baseline model + individual comorbidity (selected) (binary representation) | 0.639 (0.633-0.645) | 52091 | 0.673 (0.665-0.680) | 37462 |
| (vi) Baseline model + comorbidity using ICI (integer value of actual weight) | 0.638 (0.632-0.644) | 52121 | 0.671 (0.664-0.678) | 37505 |
| (vii) Baseline model + comorbidity using CCI weights | 0.633 (0.626-0.638) | 52307 | 0.663 (0.656-0.670) | 37666 |
| (viii) Baseline model + comorbidity using Quan weights | 0.631 (0.625-0.637) | 52389 | 0.661 (0.654-0.668) | 37714 |
| (ix) Baseline model + ECM | 0.641 (0.635-0.647) | 52106 | 0.676 (0.668-0.683) | 37450 |
| (vii) Baseline model + 23 comorbidities common to LOS and cost outcomes (binary representation)/Baseline model + 8 comorbidities common to readmission outcomes (binary representation) | 0.635 (0.629-0.641) | 52238 | 0.670 (0.663-0.677) | 37515 |

Note:

1. Baseline model includes age, sex, injury type, body region, geographic region and country of birth; outcome=all cause 30-day readmission (logistic

model)

2. Baseline model includes age, sex, injury severity, injury type, body region, geographic region and SEIFA deciles; outcome=non-planned 30 day

readmission (logistic model)

Note: See Table 5 for selected comorbidities for each outcome
