# Supplementary material for: Australian Injury Comorbidity Indices (AICIs) to predict burden and readmission among hospital-admitted injury patients
Source: BMC Health Serv Res. 2021 Feb 15;21:149. doi: 10.1186/s12913-021-06149-1 (PMC7885207; doi:10.1186/s12913-021-06149-1)
Supplement: Supplementary file 2 — Additional file 2: Appendix A1.1. ROC curves for overnight stay (age > = 15 years). [file 12913_2021_6149_MOESM2_ESM.docx]

# Appendix A1.1 – ROC curves for overnight stay (age >= 15 years)

Baseline model (age, sex, body region, injury-type, injury-severity, SEIFA, geographic region and country of birth)

Baseline model + at least one comorbidity

Baseline model + count of comorbidities

Baseline model + all 31 comorbidities

Baseline model + Charlson Comorbidity Index

Baseline model + updated CCI per Quan et. al. (2011)

Baseline model + Elixhauser Comorbidity Measure

Baseline model + AICI-os (24 conditions, binary representation)

Baseline model + comorbidity index with actual weights (24 conditions)

Baseline model + comorbidity index with rounded weights (24 conditions)

Baseline model + parsimonious index (23 conditions common to all three burden outcomes, binary representation)
