# Supplementary material for: Australian Injury Comorbidity Indices (AICIs) to predict burden and readmission among hospital-admitted injury patients
Source: BMC Health Serv Res. 2021 Feb 15;21:149. doi: 10.1186/s12913-021-06149-1 (PMC7885207; doi:10.1186/s12913-021-06149-1)
Supplement: Supplementary file 3 — Additional file 3: Appendix A1.2. Plots of predicted length of stay (days) vs observed (at least 1 overnight and LOS < =30). [file 12913_2021_6149_MOESM3_ESM.docx]

**Plots of predicted length of stay (days) vs observed (at least 1 overnight and LOS<=30)**

Baseline model (age, sex, injury severity, injury type, body region, SEIFA deciles and country of birth)

Baseline model + presence of at least one comorbidity

Baseline model + count of all comorbidities

Baseline model + all 31 comorbidities

Baseline model + Charlson Comorbidity Index

Baseline model + updated CCI per Quan et al. (2011)

Baseline model + Elixhauser Comorbidity Measure

Baseline model + AICI-los (binary representation, 27 conditions)

Baseline model + comorbidity index as a weighted summed score using actual weights (23 conditions)

Baseline model + comorbidity index as a weighted summed score using rounded weights (23 conditions)

Baseline model + parsimonious index (23 conditions common to burden outcomes, binary representation)
