# Supplementary material for: Australian Injury Comorbidity Indices (AICIs) to predict burden and readmission among hospital-admitted injury patients
Source: BMC Health Serv Res. 2021 Feb 15;21:149. doi: 10.1186/s12913-021-06149-1 (PMC7885207; doi:10.1186/s12913-021-06149-1)
Supplement: Supplementary file 4 — Additional file 4: Appendix A1.3. Residual plots for costs. [file 12913_2021_6149_MOESM4_ESM.docx]

**Appendix A1.3 – Residual plots for costs**

Baseline model (age, sex, injury severity, injury type, body region, SEIFA deciles, geographic region (metropolitan and rural) and country of birth)

Baseline model + presence of at least one comorbidity

Baseline model + count of all comorbidities

Baseline model + all 31 comorbidities

Baseline model + Charlson Comorbidity Index

Baseline model + Updated CCI by Quan et. al. (2011)

Baseline model + Elixhauser Comorbidity Measure

Baseline model + AICI-cost (binary representation 28 conditions)

Baseline model + comorbidity index as a weighted summed score using actual weights (26 conditions)

Baseline model + comorbidity index as a weighted summed score using rounded weights (26 conditions)

Baseline model + parsimonious index (23 conditions common to all burden outcomes, binary representation)
