# Supplementary material for: Australian Injury Comorbidity Indices (AICIs) to predict burden and readmission among hospital-admitted injury patients
Source: BMC Health Serv Res. 2021 Feb 15;21:149. doi: 10.1186/s12913-021-06149-1 (PMC7885207; doi:10.1186/s12913-021-06149-1)
Supplement: Supplementary file 6 — Additional file 6: Appendix A1.5. ROC curves for non-planned 30-day readmissions (age > = 15 years). [file 12913_2021_6149_MOESM6_ESM.docx]

# Appendix A1.5 – ROC curves for non-planned 30-day readmissions (age >= 15 years)

Baseline model (age, sex, body region, injury-type, injury-severity, geographic region and SEIFA)

Baseline model + at least one comorbidity

Baseline model + count of comorbidities

Baseline model + all 31 comorbidities

Baseline model + Charlson Comorbidity Index

Baseline model + updated CCI per Quan et al. (2011)

Baseline model + Elixhauser Comorbidity Measure

Baseline model + AICI-npr (11 conditions, binary representation)

Baseline model + comorbidity index with actual weights (11 conditions)

Baseline model + comorbidity index with rounded weights (11 conditions)

Baseline model + parsimonious index (8 conditions common to both readmission outcomes, binary representation)
