# Supplementary material for: Australian Injury Comorbidity Indices (AICIs) to predict burden and readmission among hospital-admitted injury patients
Source: BMC Health Serv Res. 2021 Feb 15;21:149. doi: 10.1186/s12913-021-06149-1 (PMC7885207; doi:10.1186/s12913-021-06149-1)
Supplement: Supplementary file 7 — Additional file 7: Appendix A2. Interaction plots. [file 12913_2021_6149_MOESM7_ESM.docx]

**Appendix A2 – Interaction plots**

## Overnight stay

## 1<=Length of stay<=30 days

## Hospital treatment cost

## All-cause 30-day readmissions

## Non-planned 30-day readmissions
