# Supplementary material for: Australian Injury Comorbidity Indices (AICIs) to predict burden and readmission among hospital-admitted injury patients
Source: BMC Health Serv Res. 2021 Feb 15;21:149. doi: 10.1186/s12913-021-06149-1 (PMC7885207; doi:10.1186/s12913-021-06149-1)
Supplement: Supplementary file 8 — Additional file 8: Appendix A3. Model performance details. [file 12913_2021_6149_MOESM8_ESM.docx]

# **Appendix A3**

# **Model performance detail**

### Section 3.2.5 – overnight stay

The AUCs were very similar for the Australian Injury Comorbidity Index for overnight stay (AICI-os) and ECM; model with ECM (0.761(CI 0.759-0.764)) and model with AICI-os (0.760 (CI 0.757-0.763)). The predictive ability of the CCI and updated CCI were weaker (AUCs of 0.752 (CI 0.749-0.754) and 0.752 (CI 0.749-0.754) respectively) than the aforementioned indices. There was no significant difference at the 5% level between the false negative (FN) rates of the AICI-os (30.3) and the ECM (30.2), while the FN rate of the CCI (31.1) was significantly higher.

### Section 3.2.6 – LOS (overnight stay patients)

Fit of the various models from best to worst was observed in the following order; ECM, followed by the Australian Injury Comorbidity Index for LOS (AICI-los) with twenty-seven comorbidities while the CCI and updated CCI had poorer fit. In terms of predictive ability (using the highest adjusted R^2^), the models containing the ECM (8.9%) and the AICI-los (8.8%) were higher than the CCI (8.3%) and updated CCI (8.2%), but the differences were relatively small.

Section 3.2.8 - All-cause 30-day readmission

There was no significant difference in predictive ability (based on AUC) between the model containing the Australian Injury Comorbidity Index for all-cause 30-day readmissions (AICI-acr) (AUC 0.625 (CI 0.621-0.630)), the ECM (AUC 0.626 (CI 0.622-0.631), CCI (AUC 0.622 (CI 0.617-0.626)) or updated CCI (AUC 0.620 (CI 0.615-0.624)). There was no statistically significant difference between the models with AICI-acr (41%), CCI (41%) or ECM (40.7%) with regard to FN rates.

### Section 3.2.9 - Non-planned 30-day readmissions

There were no significant differences between the AUC statistics for all the models including the Australian Injury Comorbidity Index for non-planed 30-day readmissions (AICI-npr) (AUC 0.637 (CI 0.632-0.642)), the ECM (AUC 0.639 (CI 0.634-0.644)), the CCI (AUC 0.633 (CI 0.628-0.638) and updated CCI (AUC 0.631 (CI 0.626-0.637)); the CIs overlapped. There were also no significant differences between the FN rates for the models with the AICI-npr (39.8%), CCI (40.6%) and ECM (39.8%).
