# Supplementary material for: Australian Injury Comorbidity Indices (AICIs) to predict burden and readmission among hospital-admitted injury patients
Source: BMC Health Serv Res. 2021 Feb 15;21:149. doi: 10.1186/s12913-021-06149-1 (PMC7885207; doi:10.1186/s12913-021-06149-1)
Supplement: Supplementary file 9 — Additional file 9: Appendix A4. Internal and external validation details. [file 12913_2021_6149_MOESM9_ESM.docx]

**Appendix A4**

# Internal validation

All model fit results in Appendix table A4 and false negative (FN) rates in Table 5.

## Overnight stay

All indices added with the baselines had good discrimination (AUC>= 0.7) for all subgroups, except for penetrating trauma (AUCs<0.7). The predictive power in terms of AUCs were the same for the AICI-os, AICI-b and ECM across all subgroups. The best discrimination was seen for the older adults (>65 years) and adults with intracranial injuries. The ability to discriminate was significantly poor for the CCI among older adults, males, females, non-severe injury patients and hip fractures (CIs of the CCI did not overlap with the CIs of the AICIs or ECM). The FNs for the AICI-os were significantly lower than the for the CCI when validated among children, older adults, males, females, patients with non-severe injuries, hip fractures and blunt trauma. The AICI-os also reported significantly lower FNs than the ECM among children. In all, the new indices validated better than the CCI among *all* and the ECM among *certain* subgroups.

## LOS (for patients with at least one overnight stay)

All subgroups’ validations returned adjusted R^2^s lesser than the original (full dataset) for all indices, except for the male subgroup. For children, the R^2^s were half the original but fared better than the older adults whose R^2^ was around 20-30% of the original. Validation was poorest among those with hip fractures, while non-severe injury and intracranial injury patient groups returned lesser than but close to the original R^2^s. Validations returned better R^2^s for blunt over penetrating trauma and for males than females. The new indices and the ECM had better model fit and higher predictive power in all subgroups compared to the CCI. ECM validates better than the new indices at the cost of an extra three to seven conditions.

## Cost

Using the adjusted R^2^ as a comparison measure, all indices validated a little poorly among children, males, non-severe injury and penetrating injury patients compared to the whole dataset. They validated better among older adults, females, intracranial and blunt injury patients compared with the entire dataset. The new indices outperformed the CCI on average by about three percentage points in all subgroups except among children, whilst among hip fracture patients the AICI-cost outperformed the ECM as well.

## All-cause 30-day readmission

All subgroups presented similar predictive ability in terms of the AUC statistics for all indices with all CIs overlapping. There was no significant difference between the FN rates for all indices across sub groups except that they were significantly lower for the ECM among children, older adults, females and blunt trauma patients. In all, the model nor the new or existing indices provide enough power to predict this outcome.

## Non-planned 30-day readmission

Similar to all-cause readmissions, all subgroups presented similar predictive ability in terms of the AUC statistics for all indices with all CIs overlapping. Significant differences for FN rates were only seen between the following; ECM lower than the AICI-npr and CCI among older adults, males, females, patients with hip fractures and blunt trauma while the ECM was higher than the AICI-npr and CCI for children. The AICI-npr also had a lower FN rate than the CCI for penetrating trauma. In this instance, though the ECM was better suited for certain subgroups, the overall power to predict was insufficient in this model.

# External validation

The NSW and WA validation cohorts presented similar patterns to the Victorian cohort in terms of demographics, injury characteristics and comorbidity (Table 1 and Appendix Table A1). The only differences were; (1) the overall number of patients in groups varied (which is in relation to the size of the state populations), (2) WA cohort had a smaller proportion (around one-fifth) of older adults admitted compared to the other two states (around one-third) and (3) the median length of stay for those with at least one comorbidity in the WA cohort was half of that in the other two states (2 (IQR 1-10)).

All model fit results in Appendix Table A6 and FNs in Table 5.

## Overnight stay

The predictive abilities in terms of the AUC statistics were good for all indices (AUC>0.7) and significantly higher in NSW than WA, but both lower than Victoria. They were similar for the AICI-os and ECM (CIs were overlapping), but significantly lower for the CCI. There were no significant differences between the FN rates of the AICI-os, ECM and CCI in both the NSW and WA cohorts. All indices validated well with the new indices having an advantage over the CCI in terms of predictive power and over the ECM in terms of a lesser number of comorbidities.

## LOS (for patients with at least one overnight stay)

All indices validated best in WA, followed by Victoria and NSW in terms of highest adjusted R^2^s. Highest R^2^s were for the ECM, followed by AICI-los and CCI.

## All-cause and non-planned 30-day readmission

As in the case of Victoria, the AUC statistics for modelling the new and existing indices with readmissions reveal poor predictive powers in NSW and WA as well (AUC<0.7). Apart from having low AUCs, the CIs overlap between the AICI-acr, CCI and ECM implying they are not significantly different in terms of predictive powers. Model fit was best in the ECM followed by AICI-acr and CCI (using the AIC). All-cause readmissions validated best in WA data, followed by Victoria and NSW and non-planned readmissions validated best in WA data, followed by NSW and Victoria. Overall, the model needs improvement regardless of the comorbidity index given that the AUCs lie below 0.7.
