# Supplementary material for: Australian Injury Comorbidity Indices (AICIs) to predict burden and readmission among hospital-admitted injury patients
Source: BMC Health Serv Res. 2021 Feb 15;21:149. doi: 10.1186/s12913-021-06149-1 (PMC7885207; doi:10.1186/s12913-021-06149-1)
Supplement: Supplementary file 10 — Additional file 10: Appendix A5. Using the Australian Injury Comorbidity Indices. [file 12913_2021_6149_MOESM10_ESM.docx]

# Appendix A5 – Using the Australian Injury Comorbidity Indices

The Australian Injury Comorbidity Indices for burden and readmission are recommended for use in epidemiological research where LOS, cost, all-cause and non-planned readmissions are outcomes of interest.

Option I

Model the presence of each comorbidity as a dummy variable with 1 for disease presence and 0 for absence along with other main effects. The index for LOS was derived to aid outcome prediction for up to 30 days stay while the readmissions indices were for readmissions within 30 days of discharge. Note that the conditions included in the indices vary with the outcome, unless the more parsimonious index is used.

Option II

Another option is to use a score derived by adding the presence/absence of conditions for each index as shown in Table A8.1. The total score derived is used as the comorbidity indicator. Table A8.2 shows the scores for the Victorian study population based on percentiles.

In prediction modelling, this option results in similar predictive power for all outcomes but higher false negative rates for overnight stay compared to option I.

Option II would be more useful in a clinical setting where patient classification is required, in the absence of retrospective data. The use of this score would need significant validations before it can be reliably used.

Table A8.1: Index calculation for a general injury patient cohort

| Comorbidity | AICI-os | AICI-los | AICI-cost | AICI-b | AICI-acr | AICI-npr | AICI-r |
| --- | --- | --- | --- | --- | --- | --- | --- |
| HIV/AIDS |  |  |  |  |  |  |  |
| Alcohol dependence | 1 | 1 | 1 | 1 | 1 | 1 | 1 |
| Drug dependence | 1 | 1 | 1 | 1 |  | 1 |  |
| Any malignancy | 1 | 1 | 1 | 1 | 1 |  |  |
| Blood loss anaemia | 1 | 1 | 1 | 1 |  |  |  |
| Cardiac arrhythmia | 1 | 1 | 1 | 1 |  |  |  |
| Cerebrovascular disease |  | 1 |  |  |  |  |  |
| Chronic pulmonary disease | 1 | 1 | 1 | 1 | 1 | 1 | 1 |
| Coagulopathy | 1 | 1 | 1 | 1 | 1 | 1 | 1 |
| Congestive heart failure | 1 | 1 | 1 | 1 |  | 1 |  |
| Deficiency anaemias | 1 | 1 | 1 | 1 |  |  |  |
| Dementia | 1 |  | 1 |  |  |  |  |
| Depression | 1 | 1 | 1 | 1 | 1 |  |  |
| Diabetes with chronic complications | 1 | 1 | 1 | 1 | 1 | 1 | 1 |
| Diabetes without complications | 1 | 1 | 1 | 1 | 1 | 1 | 1 |
| Hemiplegia/paraplegia | 1 | 1 | 1 | 1 |  |  |  |
| Hypertension complicated |  |  | 1 |  |  |  |  |
| Hypertension uncomplicated | 1 | 1 | 1 | 1 |  |  |  |
| Hypothyroidism | 1 | 1 | 1 | 1 |  |  |  |
| Metastatic solid tumor |  | 1 |  |  |  | 1 |  |
| Mild liver disease | 1 | 1 | 1 | 1 | 1 | 1 | 1 |
| Moderate or severe liver disease | 1 | 1 | 1 | 1 |  |  |  |
| Myocardial infarction |  |  | 1 |  |  |  |  |
| Obesity | 1 | 1 | 1 | 1 |  |  |  |
| Peptic ulcer disease |  | 1 | 1 |  |  |  |  |
| Peripheral vascular disease | 1 | 1 | 1 | 1 |  |  |  |
| Psychoses | 1 | 1 | 1 | 1 | 1 | 1 | 1 |
| Pulmonary circulation disorders |  | 1 | 1 |  |  |  |  |
| Renal disease including renal failure | 1 | 1 | 1 | 1 | 1 | 1 | 1 |
| Rheumatic disease including some other connective tissue disorders | 1 | 1 | 1 | 1 |  |  |  |
| Valvular disease | 1 | 1 | 1 | 1 |  |  |  |
| Maximum total comorbidity score | 24 | 27 | 28 | 23 | 10 | 11 | 8 |

Table A8.2: Minimum and maximum scores for injury comorbidity indices at selected percentiles for the Victorian cohorts (age >15 years)

| Percentile | AICI-os | AICI-los | AICI-cost | AICI-b | AICI-acr | AICI-npr | AICI-r |
| --- | --- | --- | --- | --- | --- | --- | --- |
| 25^th^ |  |  |  |  |  |  |  |
| 50th |  |  |  |  |  |  |  |
| 75th |  |  |  |  |  |  |  |
| 0-75th |  | 0-0 |  |  |  |  |  |
| 76th |  | 1-1 |  |  |  |  |  |
| 0-76th | 0-0 |  | 0-0 |  |  |  |  |
| 77th | 1-1 |  | 1-1 |  |  |  |  |
| 0-77th |  |  |  | 0-0 |  |  |  |
| 78th |  |  |  | 1-1 |  |  |  |
| 0-82nd |  |  |  |  | 0-0 |  |  |
| 83rd |  |  |  |  | 1-1 |  |  |
| 0-83rd |  |  |  |  |  | 0-0 |  |
| 84th |  |  |  |  |  | 1-1 |  |
| 0-84th |  |  |  |  |  |  | 0-0 |
| 85th |  |  |  |  |  |  | 1-1 |
| 93rd | 2-2 | 2-2 | 2-2 |  |  |  |  |
| 94th |  |  |  | 2-2 |  |  |  |
| 97th |  |  |  |  | 2-2 | 2-2 |  |
| 98th | 3-3 | 3-3 | 3-3 | 3-3 |  |  | 2-2 |
| 99th |  |  |  |  |  |  |  |
| 100th | 4-8 | 4-9 | 4-9 | 4-8 | 3-6 | 3-5 | 3-5 |
